# Supplementary material for: How to approach machine learning-based prediction of drug/compound–target interactions
Source: J Cheminform. 2023 Feb 6;15:16. doi: 10.1186/s13321-023-00689-w (PMC9901167; doi:10.1186/s13321-023-00689-w)
Supplement: Supplementary file 1 — Additional file 1: Table S1.. Model performance scores (in terms of MCC) in the small-scale analysis (on the compound-centric datasets) for; (a) RF, and (b) SVM models. The 3 best performances for each dataset are shown in bold font. For the results based on other performance metrics please see the supplementary spreadsheets attached as external files named: “small-scale_rf_overall_test_results.xlsx” and “small-scale_svm_overall_test_results.xlsx”. Table S2. Model performance scores in the medium-scale analysis (on the mDavis dataset). The best performance for each metric is shown in bold font. Table S3. Model performance scores (in terms of the median corrected MCC) in the large-scale analysis on the protein family specific datasets of; (a) the random-split, (b) dissimilar-compound-split, and (c) the fully-dissimilar split.The 3 best performances for each protein family are shown in bold font (ran200_ran-ecfp4: random200_random-ecfp4, only-ran-ecfp4: only-random-ecfp4). For the results based on other performance metrics please see the supplementary spreadsheet attached as an external file named: “large-scale_overall_test_results.xlsx”. Table S4: Prediction error percentages of transformer-avg models with different thresholds on random, dissimilar-compound, and fully-dissimilar splits of transferases family dataset. Table S5. Statistics of the protein family specific datasets used in the large-scale analysis. Table S6. Split-based statistics of the protein family specific datasets for; (a) the random-split, (b) dissimilar-compound-split, and (c) the fully-dissimilar-split sets (ratio represents the ratio of train to test). Table S7. Statistics of the compound-centric datasets used in the small-scale analysis. Table S8. Enzyme sub-classes of the protein family specific datasets used in the large-scale analysis. Figure S1. t-SNE based visualization of conventional and learned protein representations on; (a) enzymes including hydrolases, oxidoreductases, proteases, tra [file 13321_2023_689_MOESM1_ESM.pdf]

Additional file 1

How to Approach Machine Learning-based Prediction of Drug/Compound-Target Interactions

Heval Atas Guvenilir[1,2], Tunca Doğan[1,3,4*]

[1] Biological Data Science Laboratory, Department of Computer Engineering, Hacettepe University, Ankara, Turkey

[2] Department of Health Informatics, Graduate School of Informatics, METU, Ankara, Turkey

[3] Institute of Informatics, Hacettepe University, Ankara, Turkey

[4] Department of Bioinformatics, Graduate School of Health Sciences, Hacettepe University, Ankara, Turkey

* To whom correspondence should be addressed, e-mail: tuncadogan@gmail.com

Supplementary Tables

Table S1. Model performance scores (in terms of MCC) in the small-scale analysis (on the compound-centric datasets) for; **(a)** random forest, and **(b)** SVM models. The 3 best performances for each dataset are shown in bold font. For the results based on other performance metrics please see the supplementary spreadsheets attached as external files named: “small-scale_rf_overall_test_results.xlsx” and “small-scale_svm_overall_test_results.xlsx”.

(a)

Model	ChEMBL id (only the numeric part) of the center compound of each compound cluster									Mean	Standard error
	44	50	83	91	104	633	808	116438	295698		
aac	0.283	0.112	0.359	0.238	0.413	0.199	0.227	0.099	0.195	0.236	0.035
aac_pssm	0.226	0.278	0.395	0.432	0.170	0.212	0.399	0.153	0.270	0.282	0.035
aadp_pssm	0.166	0.211	0.396	0.453	0.177	0.347	0.330	0.240	0.380	0.300	0.035
aatp_pssm	0.231	0.312	0.432	0.477	0.332	0.280	0.398	0.254	0.387	0.345	0.028
ab_pssm	0.237	0.231	0.397	0.408	0.157	0.323	0.487	0.206	0.364	0.312	0.037
apaac	0.263	0.262	0.463	0.346	0.303	0.514	0.422	**0.293**	0.384	0.361	0.030
cksaagp	0.243	0.034	0.390	0.333	0.222	0.323	0.261	0.120	0.260	0.243	0.037
cksaap	0.324	0.268	0.510	0.410	0.297	0.489	0.405	0.143	0.431	0.364	0.039
ctdc	0.117	-0.035	0.361	0.213	0.332	0.280	0.223	0.185	0.109	0.199	0.041
ctdd	0.176	0.209	0.446	0.204	0.142	0.512	0.471	0.185	0.390	0.304	0.049
ctdt	0.212	0.033	0.309	0.201	0.363	0.169	0.379	0.130	0.301	0.233	0.038
ctriad	0.231	0.179	0.355	0.230	0.243	0.449	0.285	0.280	0.396	0.294	0.029
d_fpssm	**0.364**	0.288	0.429	0.371	0.398	0.518	0.492	0.250	0.345	0.384	0.029
dde	0.302	0.293	**0.528**	**0.492**	0.356	0.512	**0.536**	0.174	0.446	**0.404**	0.043
dp_pssm	0.279	**0.376**	0.369	0.387	0.297	0.316	0.325	0.278	0.396	0.336	0.016
dpc	0.318	0.170	0.467	0.482	0.197	0.453	0.395	0.121	0.412	0.335	0.046
dpc_pssm	0.227	0.210	0.396	0.491	0.198	0.369	0.360	0.233	0.433	0.324	0.036
edp_pssm	0.144	0.172	0.375	0.388	0.164	0.141	0.358	0.152	0.273	0.241	0.036
eedp_pssm	0.186	0.225	0.419	0.433	0.164	0.310	0.336	0.022	0.262	0.262	0.043
gaac	0.089	-0.081	0.266	0.020	0.088	0.200	0.135	-0.057	0.129	0.088	0.038
gdpc	0.314	0.045	0.311	0.292	0.253	0.310	0.262	0.076	0.232	0.233	0.034
geary	0.337	0.182	0.390	0.394	0.294	0.339	0.360	**0.345**	0.322	0.329	0.021
gtpc	0.304	0.134	0.344	0.198	0.122	0.442	0.339	0.184	0.404	0.275	0.039
k-sep_pssm	0.330	0.312	**0.561**	0.326	**0.452**	0.506	0.313	0.209	0.355	0.374	0.037
ksctriad	0.295	0.190	0.394	0.268	0.316	0.448	0.315	0.172	0.387	0.310	0.031
medp_pssm	0.167	0.164	0.417	0.431	0.216	0.309	0.375	0.023	0.285	0.265	0.045
moran	0.325	0.236	0.367	0.391	0.264	0.412	0.415	0.247	0.377	0.337	0.024
nmbroto	0.254	0.190	0.365	0.444	0.277	0.327	0.344	0.195	0.391	0.310	0.029
paac	0.230	0.181	0.502	0.417	0.255	0.505	0.500	0.262	0.347	0.356	0.043
pfam	0.325	0.286	0.486	0.432	0.430	0.203	0.273	0.132	0.399	0.329	0.039
pse_pssm	0.209	0.267	0.438	0.327	0.386	0.228	0.340	**0.314**	0.396	0.323	0.026
pssm_ac	**0.371**	**0.379**	0.393	0.426	0.324	**0.578**	0.451	0.210	**0.478**	**0.401**	0.034
pssm_cc	0.293	0.276	0.401	0.413	0.302	0.483	0.400	0.274	0.398	0.360	0.025
pssm_composition	0.220	0.221	0.455	0.451	0.333	0.373	**0.507**	0.142	0.437	0.349	0.043
qso	0.229	0.247	0.410	0.224	0.378	0.433	0.291	0.141	0.348	0.300	0.033
random200	0.071	0.080	-0.035	-0.033	-0.046	-0.100	-0.055	-0.111	0.054	-0.019	0.024
rpm_pssm	0.289	0.132	0.401	0.471	**0.440**	0.410	0.358	0.189	0.422	0.346	0.039
rpssm	0.055	0.197	0.355	0.456	0.187	0.340	0.418	0.087	0.338	0.270	0.048
spmap	0.338	0.259	0.431	0.405	0.194	**0.556**	0.489	0.153	**0.457**	0.365	0.046
taap	0.174	0.179	0.455	0.283	**0.431**	0.442	0.402	0.102	0.309	0.309	0.044
tpc	0.245	0.268	0.476	**0.499**	0.270	0.529	**0.528**	0.124	**0.448**	0.376	0.050
tpc_pssm	0.220	0.263	0.330	**0.492**	0.352	0.241	0.356	0.241	0.412	0.323	0.030
tri-gram_pssm	**0.350**	**0.349**	**0.541**	0.362	0.426	**0.545**	0.386	0.274	0.352	**0.398**	0.030

(b)

Model	ChEMBL id (only the numeric part) of the center compound of each compound cluster									Mean	Standard error
	44	50	83	91	104	633	808	116438	295698		
aac	0.122	0.049	0.183	0.102	0.243	0.279	0.091	0.161	0.136	0.152	0.025
aac_pssm	0.178	0.146	0.396	**0.488**	0.285	0.306	0.378	0.144	0.242	0.285	0.040
aadp_pssm	0.195	0.142	0.360	0.287	0.218	0.254	0.394	0.132	0.359	0.260	0.032
aatp_pssm	0.096	0.180	0.390	0.392	0.311	0.305	0.471	0.132	0.262	0.282	0.042
ab_pssm	0.296	0.311	0.385	0.372	0.201	0.470	0.370	0.195	0.295	0.322	0.030
apaac	0.159	0.292	**0.508**	0.430	0.358	**0.559**	0.438	0.200	0.300	0.360	0.045
cksaagp	0.215	0.045	0.238	0.248	0.240	0.216	0.146	0.229	0.283	0.207	0.024
cksaap	0.329	0.260	0.336	0.380	0.433	0.316	**0.491**	0.164	**0.506**	0.357	0.036
ctdc	0.197	-0.055	0.280	0.225	0.353	0.158	0.134	0.079	0.121	0.166	0.039
ctdd	0.151	0.226	0.375	0.357	0.225	0.310	0.414	0.237	0.024	0.258	0.041
ctdt	0.229	-0.013	0.233	0.099	**0.498**	0.300	0.284	0.096	0.053	0.198	0.052
ctriad	0.171	0.174	0.293	0.215	0.408	0.329	0.233	0.164	0.271	0.251	0.027
d_fpssm	0.205	0.123	0.242	0.388	0.025	0.140	0.394	0.033	0.362	0.212	0.048
dde	0.307	0.301	0.360	0.283	0.389	0.386	0.358	0.238	0.409	0.337	0.019
dp_pssm	0.228	**0.434**	0.370	0.363	0.229	0.329	0.335	0.186	0.313	0.310	0.027
dpc	0.202	0.106	0.308	0.291	0.236	0.333	0.263	0.193	0.337	0.252	0.025
dpc_pssm	0.195	0.142	0.418	0.287	0.218	0.254	0.394	0.132	0.358	0.266	0.035
edp_pssm	0.131	0.253	0.391	0.331	0.215	0.256	0.262	0.004	0.334	0.242	0.039
eedp_pssm	0.220	0.248	0.373	0.415	0.080	0.266	**0.491**	0.059	0.283	0.271	0.048
gaac	0.151	0.067	0.172	0.120	-0.089	0.230	0.111	0.065	0.206	0.115	0.032
gdpc	0.088	0.136	0.273	0.319	0.055	0.134	0.018	0.139	0.234	0.155	0.034
geary	0.277	0.201	0.357	0.327	0.308	0.288	0.280	0.195	0.358	0.288	0.020
gtpc	0.140	0.056	0.135	0.060	0.105	0.212	0.243	**0.259**	0.362	0.175	0.034
k-sep_pssm	0.277	0.347	**0.513**	0.288	0.418	0.474	0.456	0.241	0.312	**0.370**	0.033
ksctriad	0.124	0.231	0.406	0.277	0.031	0.402	0.338	0.148	0.262	0.247	0.043
medp_pssm	0.198	0.270	0.365	0.415	0.080	0.280	**0.491**	0.091	0.271	0.274	0.046
moran	0.264	0.215	0.345	0.347	0.181	0.315	0.384	0.184	0.303	0.282	0.025
nmbroto	0.231	0.228	0.354	0.289	0.439	0.282	0.386	0.236	0.258	0.300	0.025
paac	0.257	0.268	0.480	0.314	0.246	**0.528**	0.443	0.167	0.338	0.338	0.040
pfam	0.288	0.328	0.478	**0.452**	0.327	0.353	0.432	**0.367**	0.398	**0.380**	0.021
pse_pssm	0.248	0.280	0.367	0.334	0.144	0.348	0.342	0.176	0.343	0.287	0.027
pssm_ac	0.331	**0.353**	0.290	0.392	0.351	0.423	0.357	0.164	**0.428**	0.343	0.027
pssm_cc	0.294	**0.368**	0.484	0.311	**0.517**	0.446	0.373	0.192	0.349	**0.370**	0.034
pssm_composition	0.154	0.204	0.386	0.411	0.257	0.419	0.376	0.114	0.348	0.297	0.039
qso	0.220	0.078	0.249	0.211	0.346	0.268	0.073	0.213	0.113	0.197	0.031
random200	-0.044	-0.054	-0.110	-0.206	-0.046	-0.083	0.248	-0.140	0.205	-0.026	0.051
rpm_pssm	0.331	0.177	0.376	0.426	0.300	0.430	0.435	0.062	0.377	0.324	0.042
rpssm	0.133	0.312	0.260	**0.431**	0.249	0.305	0.361	0.076	0.361	0.276	0.038
spmap	**0.342**	0.260	0.486	0.254	0.249	**0.492**	0.394	0.026	0.370	0.319	0.048
taap	0.211	0.284	0.456	0.325	0.210	0.408	0.337	0.167	0.289	0.299	0.032
tpc	**0.356**	0.218	0.417	0.354	0.300	0.419	0.269	0.239	0.418	0.332	0.026
tpc_pssm	0.111	0.260	0.285	0.232	0.237	0.276	0.264	0.209	**0.425**	0.256	0.027
tri-gram_pssm	**0.340**	0.309	**0.487**	0.368	**0.444**	0.485	0.490	**0.284**	0.331	**0.393**	0.028

Table S2. Model performance scores in the medium-scale analysis (on the mDavis dataset). The best performance for each metric is shown in bold font.

**Model**	**RMSE**	**Spearman**	**F1-score**	**MCC**
seqvec	**0.794**	**0.571**	0.530	**0.445**
k-sep_pssm	0.817	0.545	**0.531**	0.434
unirep1900	0.823	0.541	0.510	0.418
apaac	0.831	0.532	0.519	0.418
unirep5700	0.831	0.531	0.506	0.412
transformer-avg	0.839	0.519	0.508	0.410
transformer-pool	0.840	0.515	0.506	0.412
qso	0.843	0.519	0.486	0.384
dde	0.845	0.508	0.480	0.384
geary	0.847	0.519	0.473	0.377
protvec	0.850	0.503	0.506	0.403
ctdd	0.851	0.503	0.484	0.376
ctriad	0.852	0.508	0.476	0.387
pfam	0.854	0.497	0.538	0.410
taap	0.863	0.492	0.467	0.349
spmap	0.871	0.491	0.477	0.362
random200	0.957	0.403	0.368	0.251
random200_random-ecfp4	0.968	0.388	0.346	0.235

Table S3. Model performance scores (in terms of the median corrected MCC) in the large-scale analysis on the protein family specific datasets of; **(a)** the random-split, **(b)** dissimilar-compound-split, and **(c)** the fully-dissimilar-split. The 3 best performances for each protein family are shown in bold font (ran200_ran-ecfp4: random200_ random-ecfp4, only-ran-ecfp4: only-random-ecfp4). For the results based on other performance metrics please see the supplementary spreadsheet attached as an external file named: “large-scale_overall_test_results.xlsx”.

(a)

**Random-split**	**epigenetic-regulators**	**hydrolases**	**ion-channels**	**membrane-receptors**	**other-enzymes**	**oxidoreductases**	**proteases**	**transcription-factors**	**transferases**	**transporters**
apaac	**0.745**	0.755	0.697	0.689	0.754	0.692	**0.735**	0.714	0.696	0.728
ctdd	0.741	0.747	0.700	0.686	**0.757**	**0.694**	0.730	0.711	0.694	**0.732**
ctriad	0.734	0.749	0.701	0.686	0.752	**0.694**	0.731	0.706	0.694	0.726
dde	0.741	**0.756**	0.703	0.689	0.754	0.692	**0.735**	0.709	0.691	0.722
geary	0.733	**0.754**	0.701	**0.694**	0.752	0.681	**0.735**	**0.721**	0.696	0.728
k-sep_pssm	**0.757**	0.749	**0.709**	0.688	0.754	0.690	**0.735**	0.706	**0.704**	0.720
pfam	0.678	0.694	0.679	0.458	0.609	0.561	0.635	0.645	0.628	0.622
qso	0.734	**0.757**	0.700	0.685	0.754	0.690	0.733	0.704	0.691	0.728
random200	0.728	0.751	0.687	0.680	0.746	0.685	0.734	0.709	0.687	0.726
spmap	0.737	0.748	0.697	0.682	**0.757**	0.680	0.728	0.709	0.683	0.720
taap	**0.760**	0.747	**0.712**	0.687	0.750	0.693	**0.736**	**0.721**	0.700	**0.730**
protvec	0.741	0.742	0.703	**0.693**	**0.758**	**0.696**	0.733	0.714	0.696	0.726
seqvec	**0.745**	0.749	0.699	0.690	**0.757**	0.678	0.728	0.709	0.700	0.724
transformer-avg	0.736	0.748	**0.707**	**0.691**	**0.760**	0.681	0.734	0.701	0.702	0.718
transformer-pool	0.734	0.746	0.695	0.689	0.741	0.684	0.733	0.714	0.694	**0.730**
unirep1900	0.744	0.745	0.703	0.686	0.753	**0.696**	0.731	**0.716**	**0.703**	0.728
unirep5700	0.729	0.749	0.690	0.688	0.755	0.690	0.734	0.706	**0.705**	0.726
only-ecfp4	0.591	0.665	0.643	0.426	0.600	0.514	0.519	0.534	0.576	0.503
ran200_ran-ecfp4	0.382	0.481	0.400	0.256	0.449	0.401	0.320	0.265	0.319	0.235
only-ran-ecfp4	0.296	0.175	0.082	0.165	0.358	0.137	0.189	0.171	0.224	0.173

(b)

**Dissimilar-compound-split**	**epigenetic-regulators**	**hydrolases**	**ion-channels**	**membrane-receptors**	**other-enzymes**	**oxidoreductases**	**proteases**	**transcription-factors**	**transferases**	**transporters**
apaac	0.137	0.355	0.342	0.249	0.419	0.391	0.381	0.058	**0.358**	0.362
ctdd	0.021	**0.407**	0.311	0.241	0.386	0.423	**0.391**	0.048	0.342	0.405
ctriad	0.045	0.351	0.276	0.243	0.354	0.405	0.353	0.006	0.346	**0.425**
dde	0.089	0.371	0.327	0.223	0.359	0.398	0.341	0.071	0.340	0.403
geary	0.044	0.375	0.291	0.242	0.397	0.400	**0.394**	0.036	**0.347**	0.417
k-sep_pssm	0.239	**0.382**	0.419	**0.298**	0.381	**0.449**	0.354	0.071	0.318	0.368
pfam	**0.455**	0.329	**0.448**	0.146	**0.452**	0.339	0.319	**0.257**	0.308	0.366
qso	0.247	0.373	0.338	**0.278**	0.345	0.356	0.369	0.071	0.324	**0.419**
random200	0.152	**0.386**	0.273	0.266	0.348	0.409	0.341	0.103	0.341	0.388
spmap	0.158	0.351	0.289	**0.274**	0.361	0.395	0.383	0.036	0.335	0.369
taap	**0.289**	0.371	**0.434**	0.243	**0.443**	0.360	**0.398**	**0.187**	0.322	**0.438**
protvec	-0.024	0.348	**0.437**	0.222	0.363	0.381	0.366	**0.118**	0.344	0.390
seqvec	0.192	0.349	0.310	0.228	0.374	**0.435**	0.373	0.033	**0.359**	0.378
transformer-avg	0.075	0.349	0.288	0.250	**0.447**	**0.428**	**0.391**	0.043	0.328	0.390
transformer-pool	0.104	0.348	0.364	0.258	0.411	0.402	0.357	0.061	0.316	0.376
unirep1900	0.161	0.334	0.277	0.256	0.402	0.400	0.376	0.076	0.329	0.370
unirep5700	0.061	0.350	0.289	0.255	0.387	0.374	0.346	0.090	0.320	0.376
only-ecfp4	**0.428**	0.244	0.243	0.168	0.419	0.281	0.307	0.058	0.306	0.309
ran200_ran-ecfp4	-0.076	0.254	0.293	0.133	0.210	0.270	0.138	0.083	0.178	0.284
only-ran-ecfp4	0.004	-0.020	0.018	0.001	-0.028	-0.015	-0.008	0.016	-0.020	-0.035

(c)

**Fully-dissimilar-split**	**epigenetic-regulators**	**hydrolases**	**ion-channels**	**membrane-receptors**	**other-enzymes**	**oxidoreductases**	**proteases**	**transcription-factors**	**transferases**	**transporters**
apaac	0.403	0.156	0.146	0.243	0.129	-0.044	0.192	0.063	0.300	**0.240**
ctdd	0.396	0.132	-0.074	0.253	0.162	0.074	0.207	0.101	0.247	-0.017
ctriad	**0.420**	**0.203**	0.086	0.220	0.125	0.030	0.212	**0.238**	0.273	**0.276**
dde	0.375	0.170	0.124	0.206	**0.232**	-0.050	0.195	0.150	0.295	0.029
geary	0.319	0.160	0.155	0.195	0.172	0.044	**0.268**	0.066	0.275	-0.027
k-sep_pssm	0.252	0.181	0.157	0.137	0.043	0.052	-0.134	0.086	0.300	**0.297**
pfam	**0.446**	**0.208**	**0.174**	**0.270**	0.221	0.088	0.142	0.156	0.301	0.198
qso	0.397	0.111	0.044	0.166	0.187	0.141	0.215	0.129	0.300	0.202
random200	0.289	0.040	0.146	0.226	**0.282**	0.070	0.149	0.051	0.284	-0.194
spmap	0.361	0.103	**0.182**	0.213	0.114	0.015	0.209	0.091	0.287	0.118
taap	0.289	0.155	**0.181**	**0.286**	0.208	-0.028	0.205	0.129	0.310	0.194
protvec	0.275	0.146	**0.174**	0.235	0.131	0.077	0.184	**0.160**	0.301	0.204
seqvec	0.372	0.154	0.032	0.155	0.199	0.018	0.046	0.150	0.276	-0.023
transformer-avg	0.367	0.129	0.058	**0.265**	0.176	0.092	**0.227**	0.144	0.311	0.187
transformer-pool	0.403	0.148	-0.052	0.244	0.079	**0.133**	0.170	0.117	**0.313**	-0.040
unirep1900	0.325	0.186	0.143	0.217	0.205	**0.132**	0.159	-0.008	**0.332**	0.004
unirep5700	0.339	0.154	0.143	0.204	0.229	**0.226**	0.189	0.127	**0.324**	0.114
only-ecfp4	**0.426**	**0.205**	0.133	0.254	**0.240**	0.016	**0.287**	**0.169**	0.294	0.172
ran200_ran-ecfp4	0.087	0.067	-0.012	0.022	-0.001	0.123	0.061	-0.003	0.057	0.032
only-ran-ecfp4	0.041	-0.003	-0.013	0.001	0.010	-0.031	-0.019	0.001	0.018	0.025

Table S4: Prediction error percentages of transformer-avg models with different thresholds on random, dissimilar-compound, and fully-dissimilar splits of transferases family dataset.

	PE > 0.5 (%)	PE > 1 (%)	PE > 1.5 (%)	PE > 2 (%)
**Random-split**	36.6	11.8	3.6	0.9
**Dissimilar-compound-split**	69.6	40.8	17.8	6.9
**Fully-dissimilar-split**	64.9	39.0	22.5	10.7

PE: Prediction error

Table S5. Statistics of the protein family specific datasets used in the large-scale analysis.

	Datapoints		Proteins		Compounds	
**Protein family**	**initial**	**discarded**	**initial**	**discarded**	**initial**	**discarded**
epigenetic regulators	19,219	1,055	125	8	10,637	276
hydrolases	71,996	9,051	623	12	56,313	7,282
ion channels	38,097	3,865	241	26	32,376	2,073
membrane receptors	220,810	14,635	640	3	133,440	8,357
other enzymes	36,857	4,007	456	9	21,428	3,372
oxidoreductases	59,023	4,545	404	2	42,338	2,895
proteases	99,100	11,839	409	20	64,688	8,369
transcription factors	25,292	216	119	0	17,244	80
transferases	191,864	31,900	1,020	39	124,266	18,963
transporters	29,165	804	190	2	18,493	523

Table S6. Split-based statistics of the protein family specific datasets for; **(a)** the random-split, **(b)** dissimilar-compound-split, and **(c)** the fully-dissimilar-split sets (ratio represents the ratio of train to test).

(a)

**Random-split**	**Bioactivity datapoints**			**Proteins**			**Compounds**		
**Protein family**	**train**	**test**	**ratio**	**train**	**test**	**ratio**	**train**	**test**	**ratio**
epigenetic-regulators	16,675	1,489	11.2	117	83	1.4	9,797	1,388	7.1
hydrolases	59,021	3,924	15.0	610	304	2.0	46,575	3,851	12.1
ion-channels	31,287	2,945	10.6	213	142	1.5	27,961	2,898	9.6
membrane-receptors	197,919	8,256	24.0	636	428	1.5	121,954	8,030	15.2
other-enzymes	29,465	3,385	8.7	442	256	1.7	16,757	3,040	5.5
oxidoreductases	52,277	2,201	23.8	402	240	1.7	38,277	2,169	17.6
proteases	83,561	3,700	22.6	389	219	1.8	54,628	3,598	15.2
transcription-factors	23,498	1,578	14.9	118	80	1.5	16,465	1,529	10.8
transferases	150,247	9,717	15.5	980	634	1.5	100,462	9,070	11.1
transporters	26,313	2,048	12.8	188	114	1.6	17,141	1,958	8.8

(b)

Dissimilar-compound-split	Bioactivity datapoints			Proteins			Compounds		
**Protein family**	**train**	**test**	**ratio**	**train**	**test**	**ratio**	**train**	**test**	**ratio**
epigenetic-regulators	16,763	1,401	12.0	117	52	2.3	9,536	825	11.6
hydrolases	58,953	3,992	14.8	597	178	3.4	45,913	3,118	14.7
ion-channels	31,329	2,903	10.8	208	69	3.0	27,656	2,647	10.4
membrane-receptors	197,652	8,523	23.2	627	252	2.5	118,914	6,169	19.3
other-enzymes	29,325	3,525	8.3	433	157	2.8	15,428	2,628	5.9
oxidoreductases	52,190	2,288	22.8	396	107	3.7	37,763	1,680	22.5
proteases	83,405	3,856	21.6	383	130	2.9	53,493	2,826	18.9
transcription-factors	23,444	1,632	14.4	116	38	3.1	16,015	1,149	13.9
transferases	150,216	9,748	15.4	956	393	2.4	98,103	7,200	13.6
transporters	26,273	2,088	12.6	182	63	2.9	16,545	1,425	11.6

(c)

Fully-dissimilar-split	Bioactivity datapoints			Proteins			Compounds		
**Protein family**	**train**	**test**	**ratio**	**train**	**test**	**ratio**	**train**	**test**	**ratio**
epigenetic-regulators	16,675	1,489	11.2	117	52	2.3	9,536	825	11.6
hydrolases	59,021	3,924	15.0	597	178	3.4	45,913	3,118	14.7
ion-channels	31,287	2,945	10.6	208	69	3.0	27,656	2,647	10.4
membrane-receptors	197,919	8,256	24.0	627	252	2.5	118,914	6,169	19.3
other-enzymes	29,465	3,385	8.7	433	157	2.8	15,428	2,628	5.9
oxidoreductases	52,277	2,201	23.8	396	107	3.7	37,763	1,680	22.5
proteases	83,561	3,700	22.6	383	130	2.9	53,493	2,826	18.9
transcription-factors	23,498	1,578	14.9	116	38	3.1	16,015	1,149	13.9
transferases	150,247	9,717	15.5	956	393	2.4	98,103	7,200	13.6
transporters	26,313	2,048	12.8	182	63	2.9	16,545	1,425	11.6

Table S7. Statistics of the compound-centric datasets used in the small-scale analysis.

Name of the center compound	ChEMBL id of the center compounds	cluster size (total # of compounds)	# of active targets	# of inactive targets
Curcumin	CHEMBL116438	1648	94	66
Tamoxifen	CHEMBL83	754	147	35
Quercetin	CHEMBL50	543	92	64
Genistein	CHEMBL44	479	102	57
Econazole	CHEMBL808	246	54	23
Levoketoconazole	CHEMBL295698	243	79	27
Amiodarone	CHEMBL633	194	73	28
Miconazole	CHEMBL91	170	49	22
Clotrimazole	CHEMBL104	52	38	37

Table S8. Enzyme sub-classes of the protein family specific datasets used in the large-scale analysis.

**Main class EC number(s) of the merged enzyme classes**	**Names of merged enzyme classes**
1	Oxidoreductases, Cytochrome P450s
2	Transferases, Kinases
3 (except 3.4)	Hydrolases, Phosphatases, Phosphodiesterases
3.4	Proteases (a.k.a. Peptidases)
All remaining	Other, Lyases

Supplementary Figures

(a)

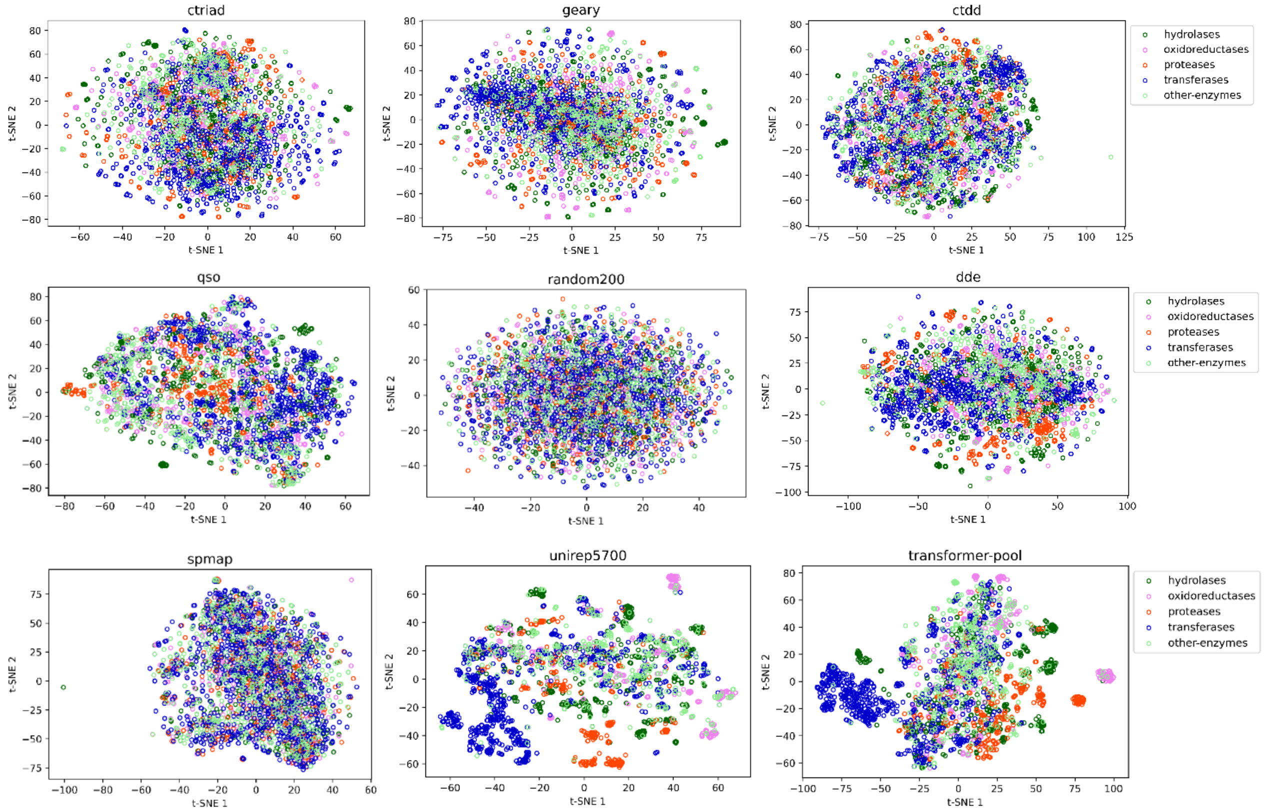

(b)

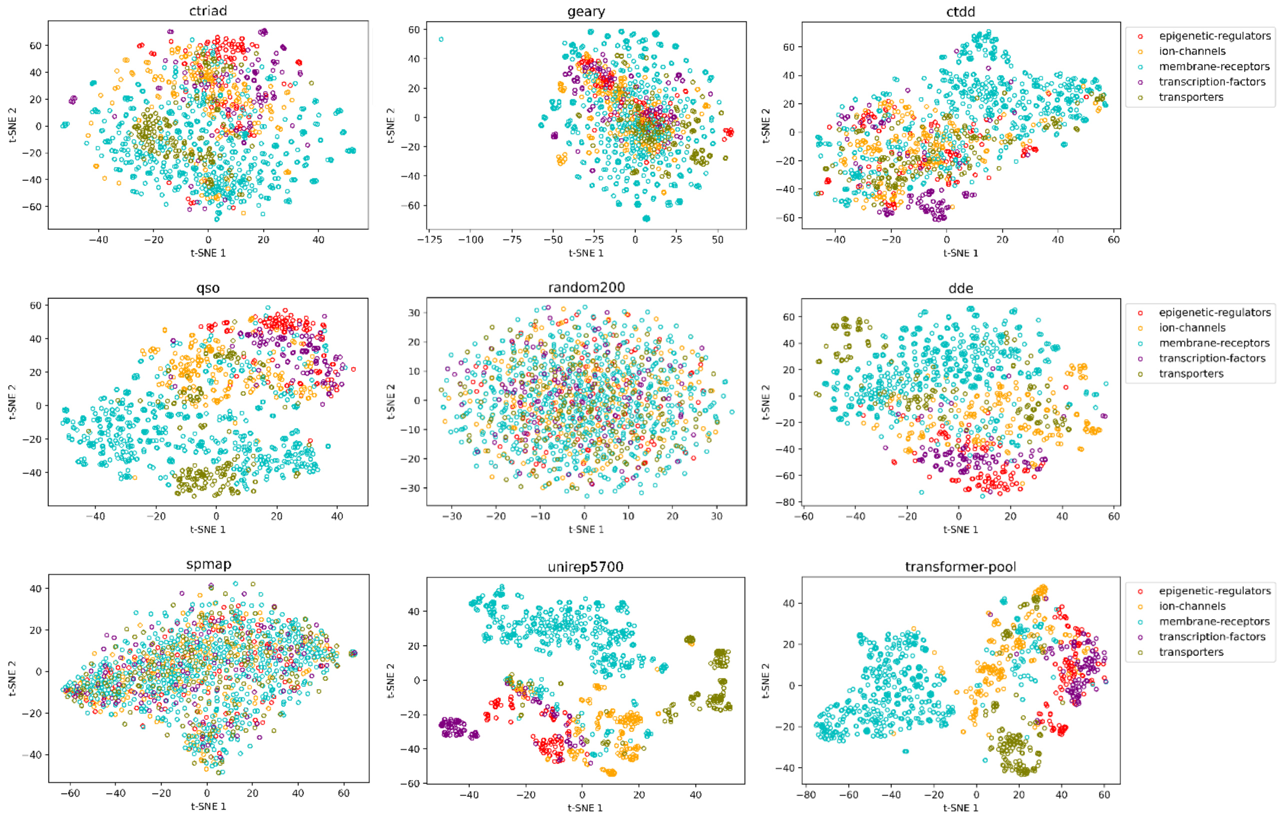

Figure S1. t-SNE based visualization of conventional and learned protein representations on; **(a)** enzymes including hydrolases, oxidoreductases, proteases, transferases, and other-enzymes, and **(b)** other protein families (non-enzymes) including epigenetic regulators, ion channels, membrane receptors, transcription factors and transporters.

(a)

Random-split

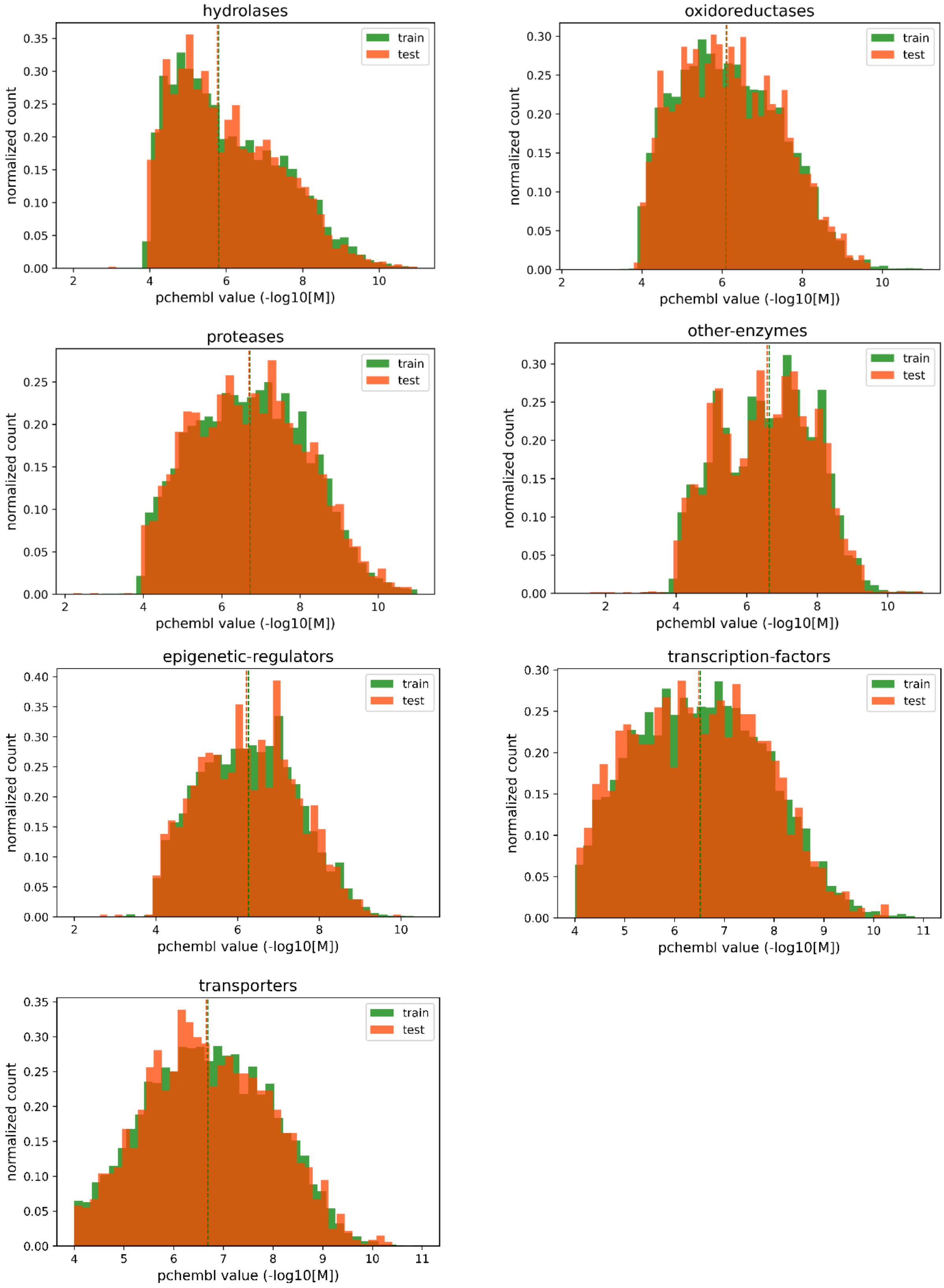

(b)

Dissimilar-compound-split

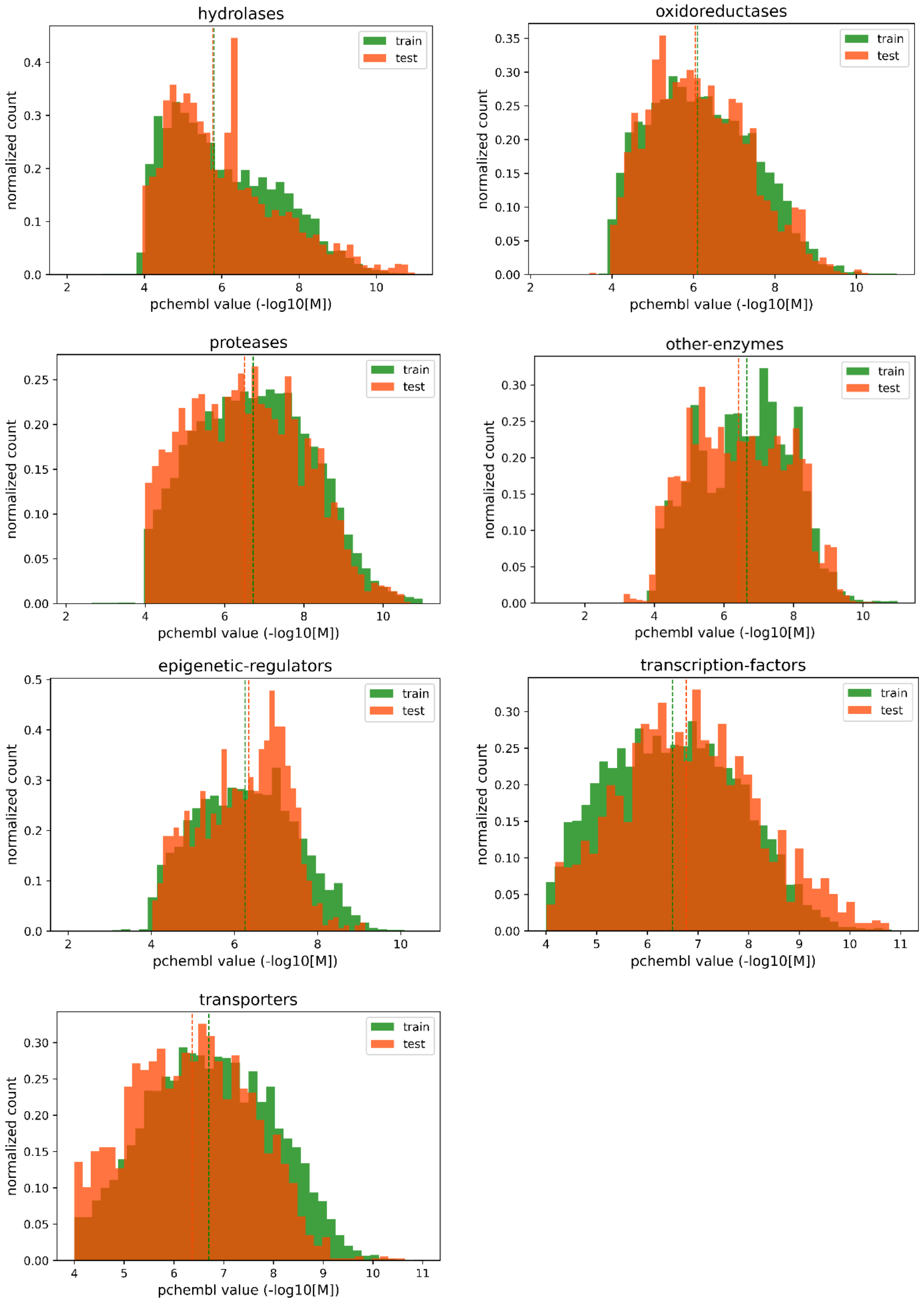

(c)

Fully-dissimilar-split

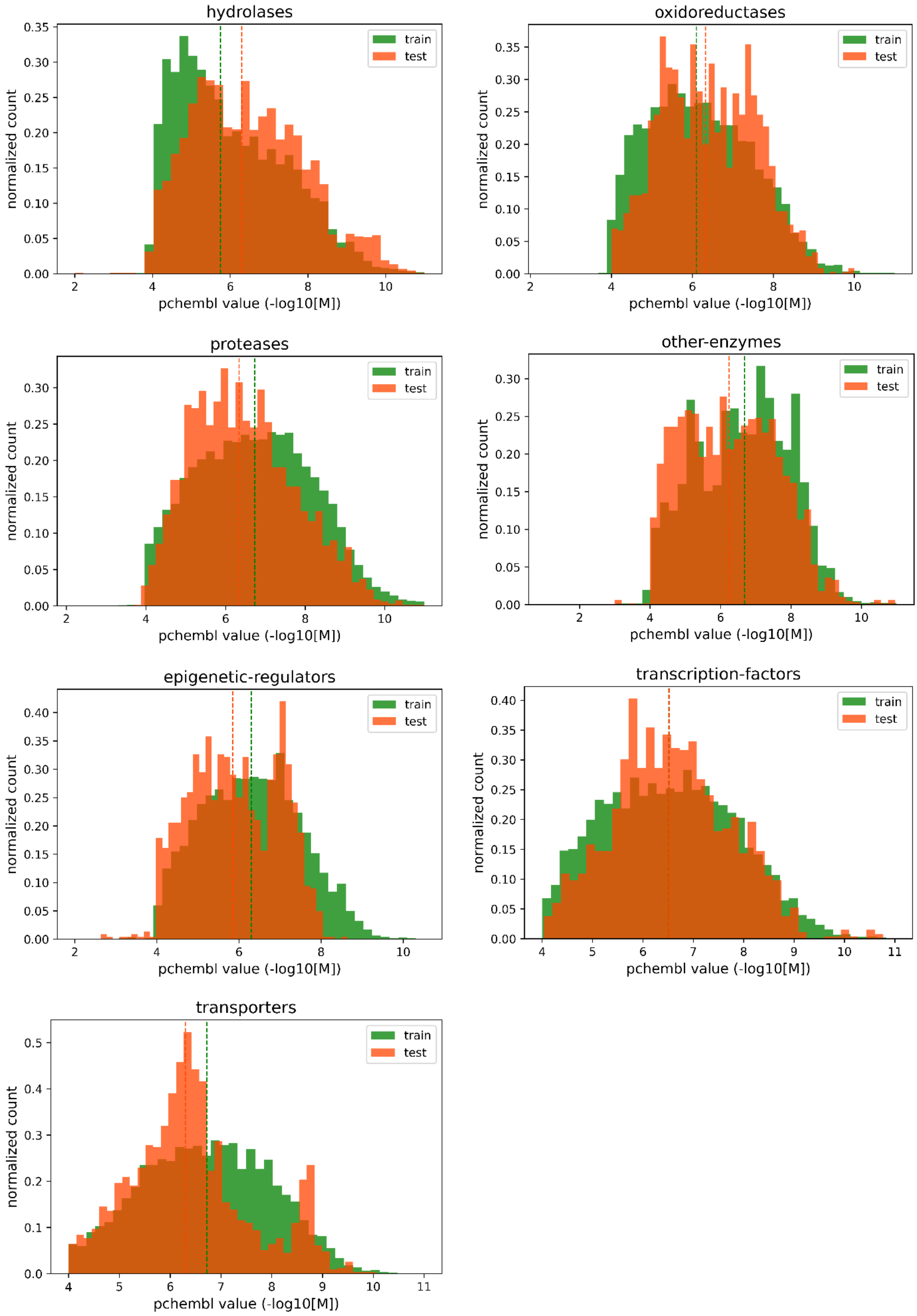

Figure S2. Bioactivity distributions of protein family-specific datasets in terms of; **(a)** random split, **(b)** dissimilar-compound split, and **(c)** fully-dissimilar split sets, together with the median values shown as vertical dashed lines.

(a) (b)

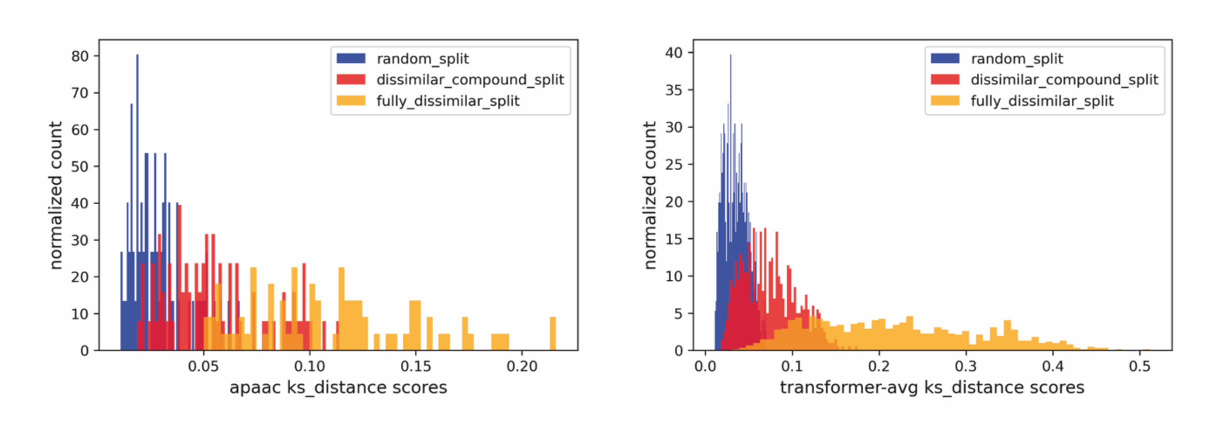

Figure S3: KS distance (between train and test samples) score distributions of **(a)** apaac, and **(b)** transformer-avg representations among random, dissimilar-compound, and fully-dissimilar splits in the transferases family proteins.

(a)

Random-split

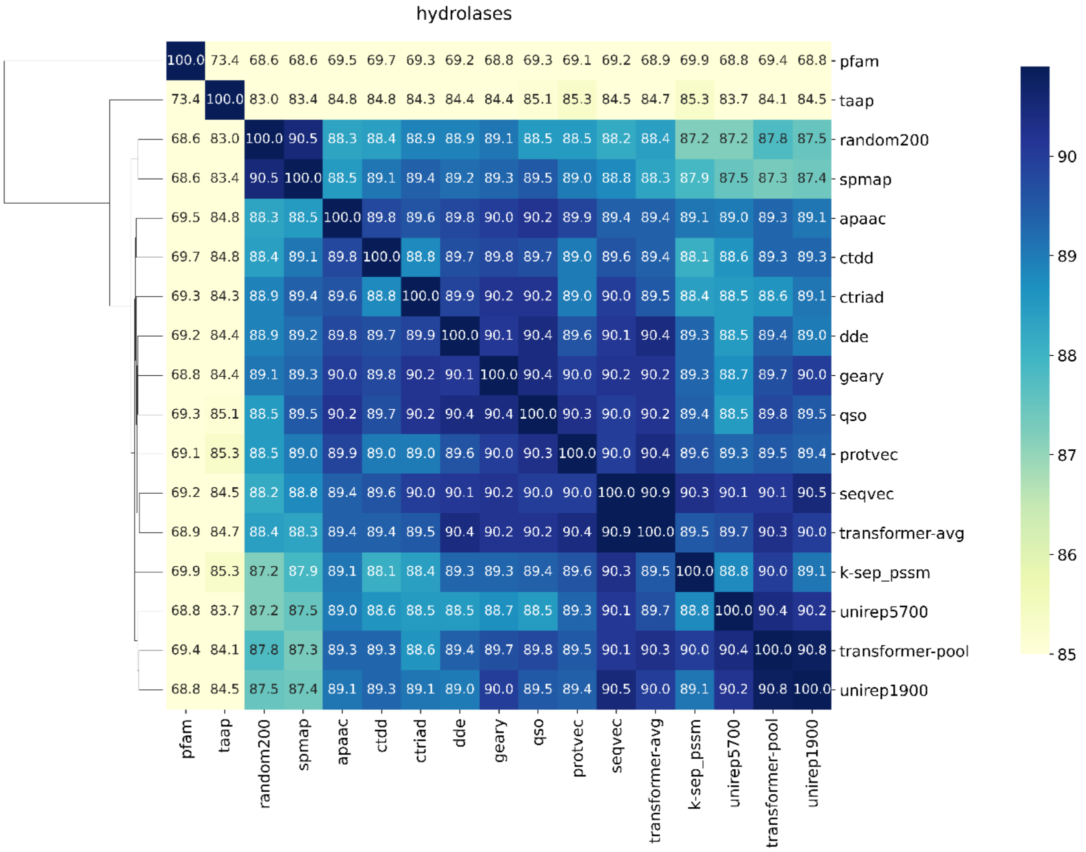

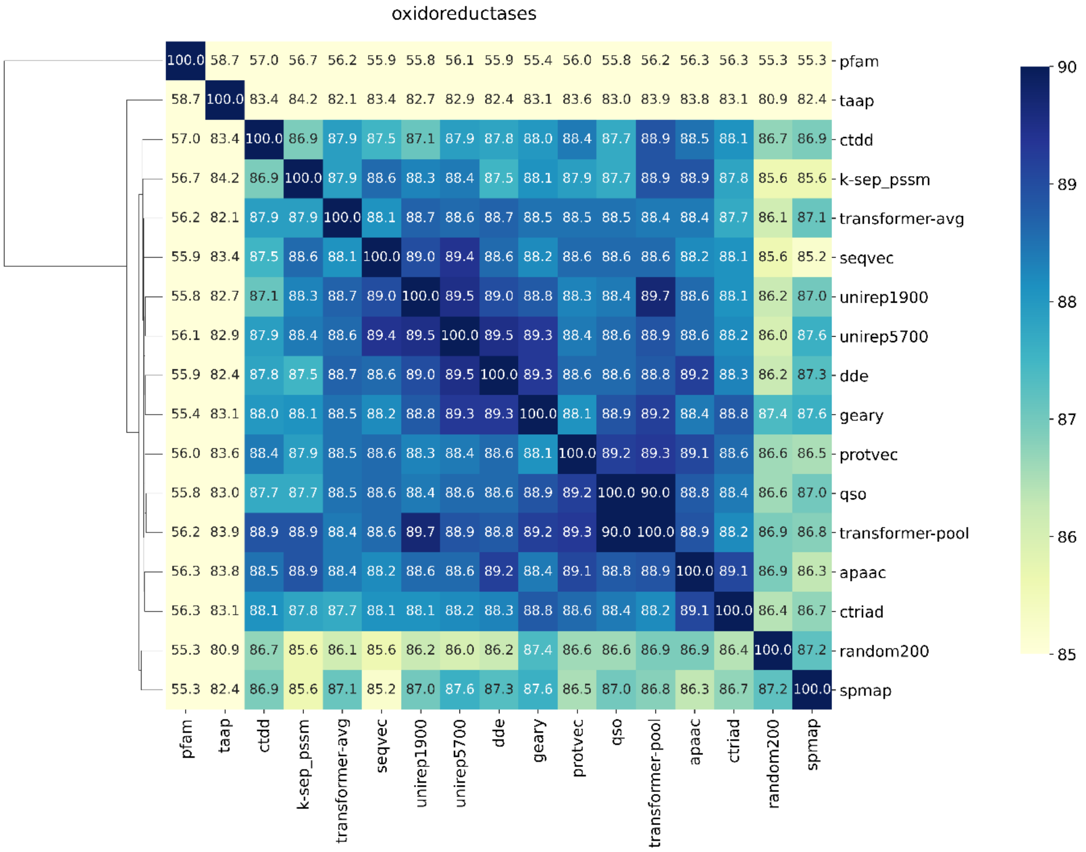

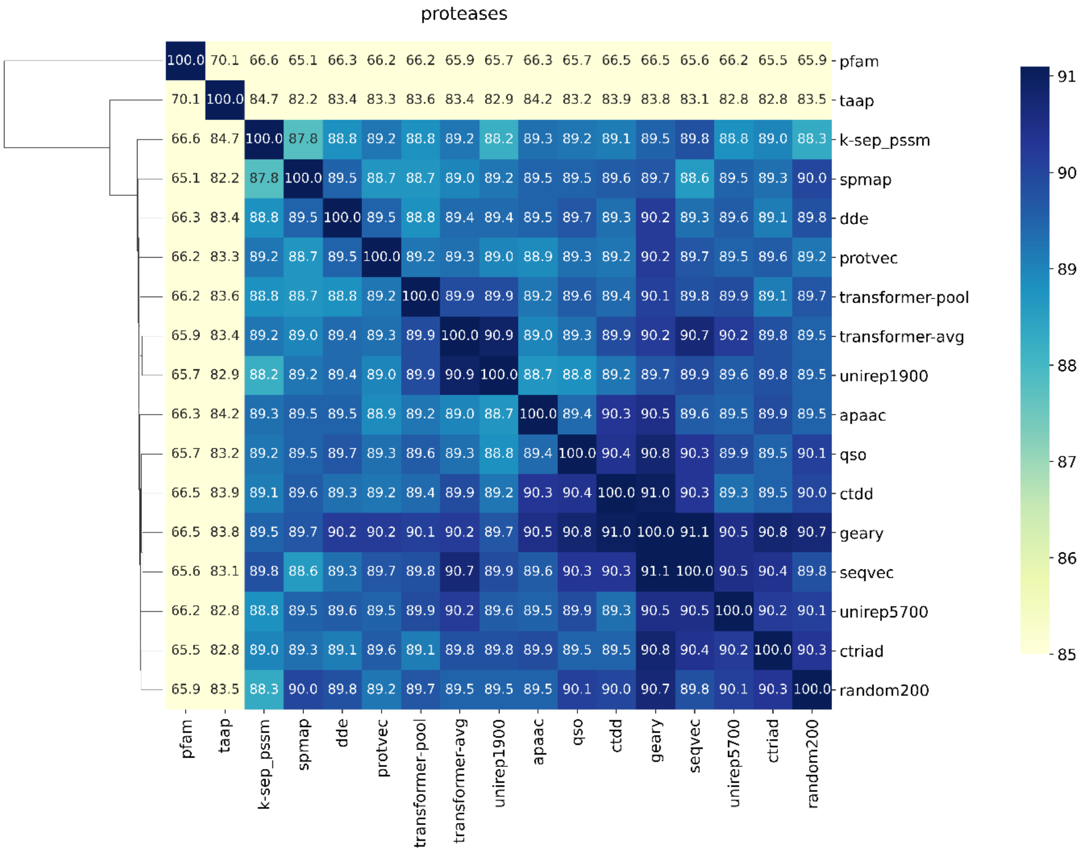
proteases
pfam
taap
k-sep_pssm
spmap
dde
protvec
transformer-pool
transformer-avg
unirep1900
apaac
qso
ctdd
geary
seqvec
unirep5700
ctriad
random200
91
90
89
88
87
86
85

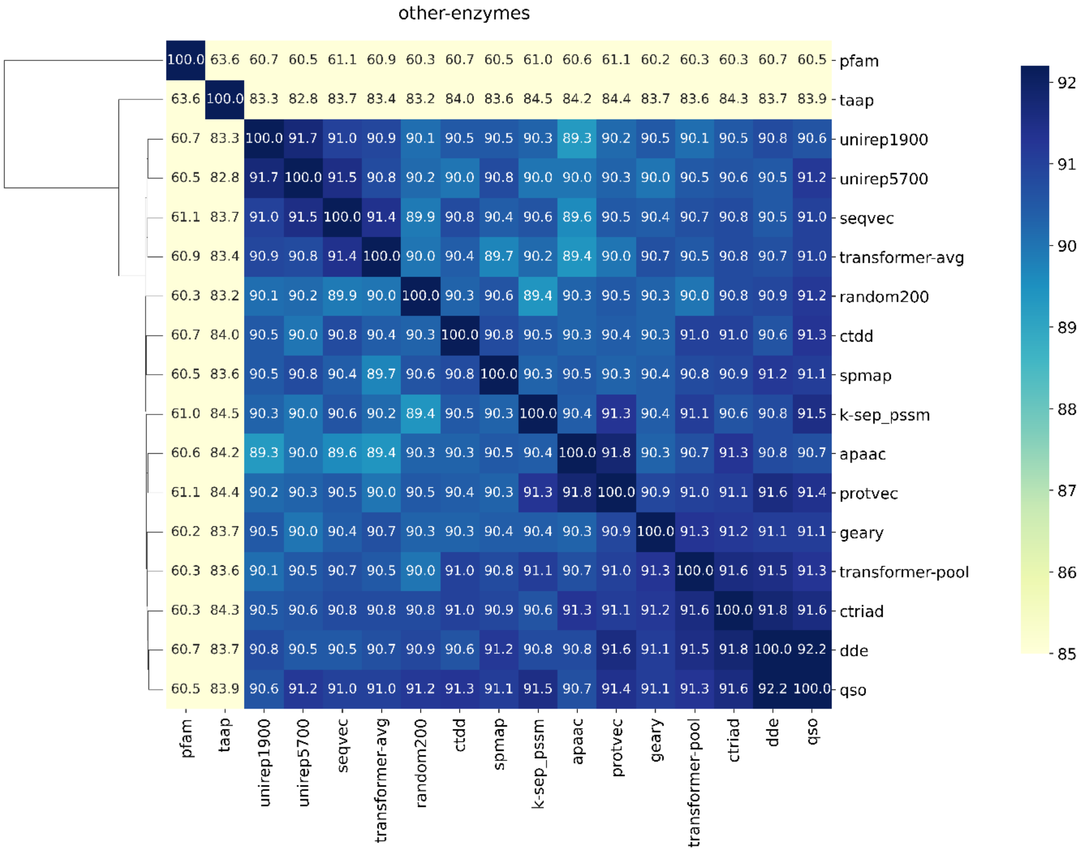
other-enzymes
pfam
taap
unirep1900
unirep5700
seqvec
transformer-avg
random200
ctdd
spmap
k-sep_pssm
apaac
protvec
geary
transformer-pool
ctriad
dde
qso
92
91
90
89
88
87
86
85

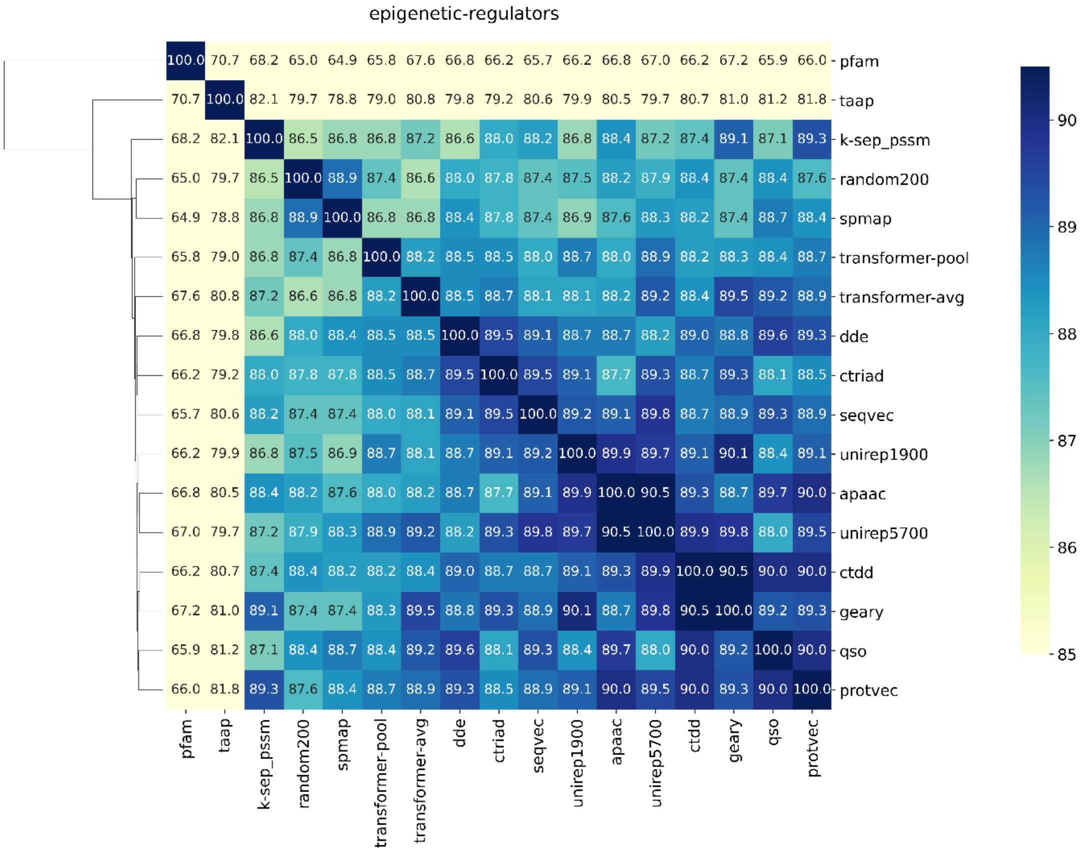
epigenetic-regulators
pfam: 100.0 70.7 68.2 65.0 64.9 65.8 67.6 66.8 66.2 65.7 66.2 66.8 67.0 66.2 67.2 65.9 66.0
taap: 70.7 100.0 82.1 79.7 78.8 79.0 80.8 79.8 79.2 80.6 79.9 80.5 79.7 80.7 81.0 81.2 81.8
k-sep_pssm: 68.2 82.1 100.0 86.5 86.8 86.8 87.2 86.6 88.0 88.2 86.8 88.4 87.2 87.4 89.1 87.1 89.3
random200: 65.0 79.7 86.5 100.0 88.9 87.4 86.6 88.0 87.8 87.4 87.5 88.2 87.9 88.4 87.4 88.4 87.6
spmap: 64.9 78.8 86.8 88.9 100.0 86.8 86.8 88.4 87.8 87.4 86.9 87.6 88.3 88.2 87.4 88.7 88.4
transformer-pool: 65.8 79.0 86.8 87.4 86.8 100.0 88.2 88.5 88.5 88.0 88.7 88.0 88.9 88.2 88.3 88.4 88.7
transformer-avg: 67.6 80.8 87.2 86.6 86.8 88.2 100.0 88.5 88.7 88.1 88.1 88.2 89.2 88.4 89.5 89.2 88.9
dde: 66.8 79.8 86.6 88.0 88.4 88.5 88.5 100.0 89.5 89.1 88.7 88.7 88.2 89.0 88.8 89.6 89.3
ctriad: 66.2 79.2 88.0 87.8 87.8 88.5 88.7 89.5 100.0 89.5 89.1 87.7 89.3 88.7 89.3 88.1 88.5
seqvec: 65.7 80.6 88.2 87.4 87.4 88.0 88.1 89.1 89.5 100.0 89.2 89.1 89.8 88.7 88.9 89.3 88.9
unirep1900: 66.2 79.9 86.8 87.5 86.9 88.7 88.1 88.7 89.1 89.2 100.0 89.9 89.7 89.1 90.1 88.4 89.1
apaac: 66.8 80.5 88.4 88.2 87.6 88.0 88.2 88.7 87.7 89.1 89.9 100.0 90.5 89.3 88.7 89.7 90.0
unirep5700: 67.0 79.7 87.2 87.9 88.3 88.9 89.2 88.2 89.3 89.8 89.7 90.5 100.0 89.9 89.8 88.0 89.5
ctdd: 66.2 80.7 87.4 88.4 88.2 88.2 88.4 89.0 88.7 88.7 89.1 89.3 89.9 100.0 90.5 90.0 90.0
geary: 67.2 81.0 89.1 87.4 87.4 88.3 89.5 88.8 89.3 88.9 90.1 88.7 89.8 90.5 100.0 89.2 89.3
qso: 65.9 81.2 87.1 88.4 88.7 88.4 89.2 89.6 88.1 89.3 88.4 89.7 88.0 90.0 89.2 100.0 90.0
protvec: 66.0 81.8 89.3 87.6 88.4 88.7 88.9 89.3 88.5 88.9 89.1 90.0 89.5 90.0 89.3 90.0 100.0
pfam taap k-sep_pssm random200 spmap transformer-pool transformer-avg dde ctriad seqvec unirep1900 apaac unirep5700 ctdd geary qso protvec
90 89 88 87 86 85

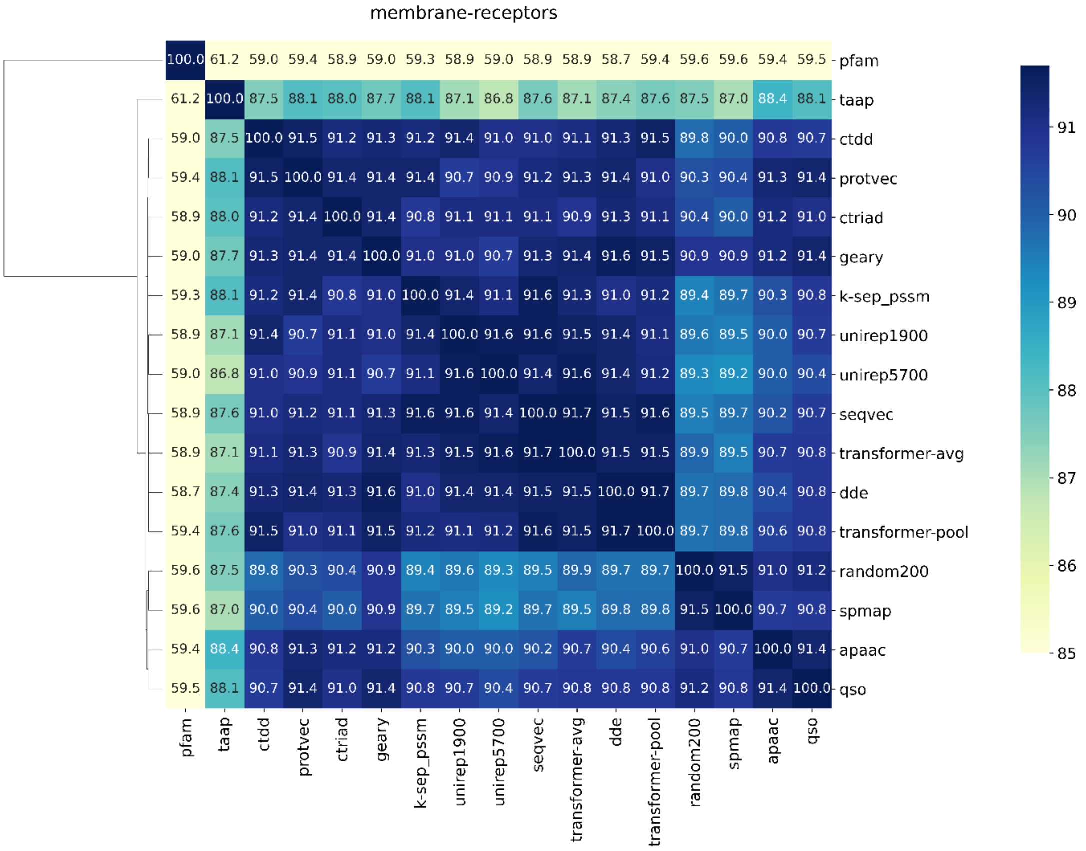
membrane-receptors
pfam: 100.0 61.2 59.0 59.4 58.9 59.0 59.3 58.9 59.0 58.9 58.9 58.7 59.4 59.6 59.6 59.4 59.5
taap: 61.2 100.0 87.5 88.1 88.0 87.7 88.1 87.1 86.8 87.6 87.1 87.4 87.6 87.5 87.0 88.4 88.1
ctdd: 59.0 87.5 100.0 91.5 91.2 91.3 91.2 91.4 91.0 91.0 91.1 91.3 91.5 89.8 90.0 90.8 90.7
protvec: 59.4 88.1 91.5 100.0 91.4 91.4 91.4 90.7 90.9 91.2 91.3 91.4 91.0 90.3 90.4 91.3 91.4
ctriad: 58.9 88.0 91.2 91.4 100.0 91.4 90.8 91.1 91.1 91.1 90.9 91.3 91.1 90.4 90.0 91.2 91.0
geary: 59.0 87.7 91.3 91.4 91.4 100.0 91.0 91.0 90.7 91.3 91.4 91.6 91.5 90.9 90.9 91.2 91.4
k-sep_pssm: 59.3 88.1 91.2 91.4 90.8 91.0 100.0 91.4 91.1 91.6 91.3 91.0 91.2 89.4 89.7 90.3 90.8
unirep1900: 58.9 87.1 91.4 90.7 91.1 91.0 91.4 100.0 91.6 91.6 91.5 91.4 91.1 89.6 89.5 90.0 90.7
unirep5700: 59.0 86.8 91.0 90.9 91.1 90.7 91.1 91.6 100.0 91.4 91.6 91.4 91.2 89.3 89.2 90.0 90.4
seqvec: 58.9 87.6 91.0 91.2 91.1 91.3 91.6 91.6 91.4 100.0 91.7 91.5 91.6 89.5 89.7 90.2 90.7
transformer-avg: 58.9 87.1 91.1 91.3 90.9 91.4 91.3 91.5 91.6 91.7 100.0 91.5 91.5 89.9 89.5 90.7 90.8
dde: 58.7 87.4 91.3 91.4 91.3 91.6 91.0 91.4 91.4 91.5 91.5 100.0 91.7 89.7 89.8 90.4 90.8
transformer-pool: 59.4 87.6 91.5 91.0 91.1 91.5 91.2 91.1 91.2 91.6 91.5 91.7 100.0 89.7 89.8 90.6 90.8
random200: 59.6 87.5 89.8 90.3 90.4 90.9 89.4 89.6 89.3 89.5 89.9 89.7 89.7 100.0 91.5 91.0 91.2
spmap: 59.6 87.0 90.0 90.4 90.0 90.9 89.7 89.5 89.2 89.7 89.5 89.8 89.8 91.5 100.0 90.7 90.8
apaac: 59.4 88.4 90.8 91.3 91.2 91.2 90.3 90.0 90.0 90.2 90.7 90.4 90.6 91.0 90.7 100.0 91.4
qso: 59.5 88.1 90.7 91.4 91.0 91.4 90.8 90.7 90.4 90.7 90.8 90.8 90.8 91.2 90.8 91.4 100.0
pfam taap ctdd protvec ctriad geary k-sep_pssm unirep1900 unirep5700 seqvec transformer-avg dde transformer-pool random200 spmap apaac qso
91 90 89 88 87 86 85

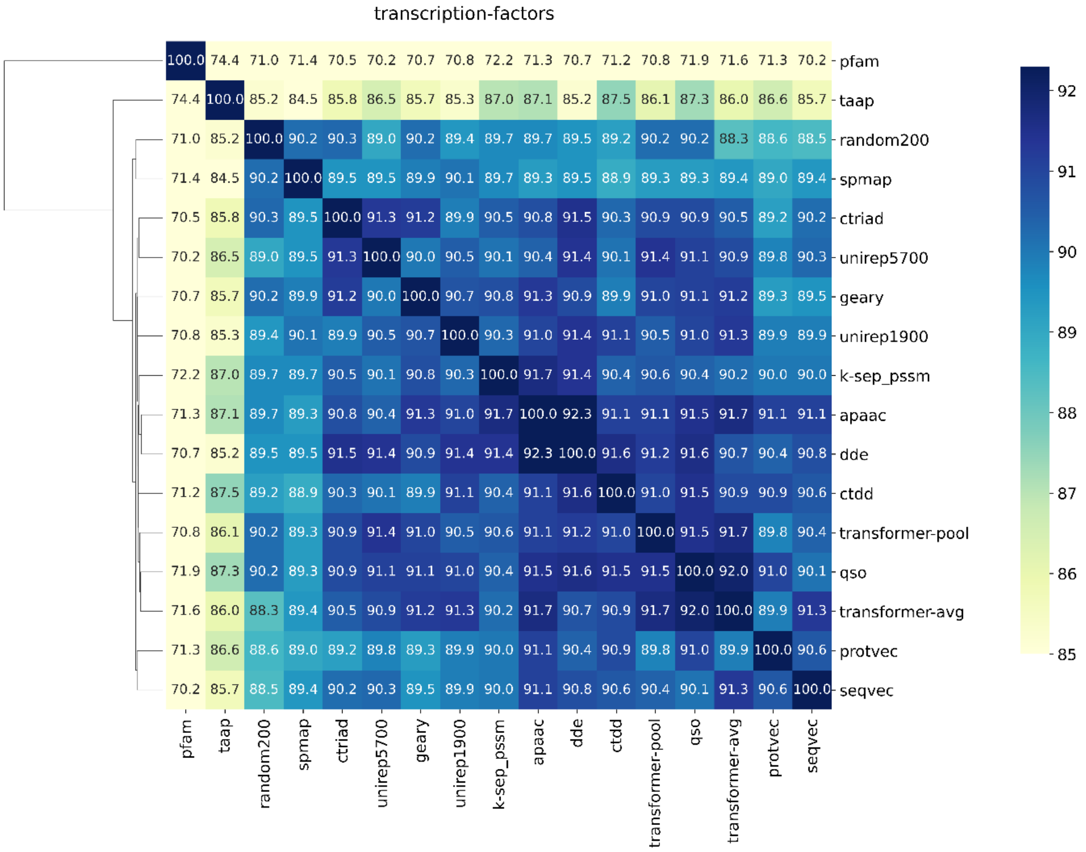
transcription-factors
pfam 100.0 74.4 71.0 71.4 70.5 70.2 70.7 70.8 72.2 71.3 70.7 71.2 70.8 71.9 71.6 71.3 70.2
taap 74.4 100.0 85.2 84.5 85.8 86.5 85.7 85.3 87.0 87.1 85.2 87.5 86.1 87.3 86.0 86.6 85.7
random200 71.0 85.2 100.0 90.2 90.3 89.0 90.2 89.4 89.7 89.7 89.5 89.2 90.2 90.2 88.3 88.6 88.5
spmap 71.4 84.5 90.2 100.0 89.5 89.5 89.9 90.1 89.7 89.3 89.5 88.9 89.3 89.3 89.4 89.0 89.4
ctriad 70.5 85.8 90.3 89.5 100.0 91.3 91.2 89.9 90.5 90.8 91.5 90.3 90.9 90.9 90.5 89.2 90.2
unirep5700 70.2 86.5 89.0 89.5 91.3 100.0 90.0 90.5 90.1 90.4 91.4 90.1 91.4 91.1 90.9 89.8 90.3
geary 70.7 85.7 90.2 89.9 91.2 90.0 100.0 90.7 90.8 91.3 90.9 89.9 91.0 91.1 91.2 89.3 89.5
unirep1900 70.8 85.3 89.4 90.1 89.9 90.5 90.7 100.0 90.3 91.0 91.4 91.1 90.5 91.0 91.3 89.9 89.9
k-sep_pssm 72.2 87.0 89.7 89.7 90.5 90.1 90.8 90.3 100.0 91.7 91.4 90.4 90.6 90.4 90.2 90.0 90.0
apaac 71.3 87.1 89.7 89.3 90.8 90.4 91.3 91.0 91.7 100.0 92.3 91.1 91.1 91.5 91.7 91.1 91.1
dde 70.7 85.2 89.5 89.5 91.5 91.4 90.9 91.4 91.4 92.3 100.0 91.6 91.2 91.6 90.7 90.4 90.8
ctdd 71.2 87.5 89.2 88.9 90.3 90.1 89.9 91.1 90.4 91.1 91.6 100.0 91.0 91.5 90.9 90.9 90.6
transformer-pool 70.8 86.1 90.2 89.3 90.9 91.4 91.0 90.5 90.6 91.1 91.2 91.0 100.0 91.5 91.7 89.8 90.4
qso 71.9 87.3 90.2 89.3 90.9 91.1 91.1 91.0 90.4 91.5 91.6 91.5 91.5 100.0 92.0 91.0 90.1
transformer-avg 71.6 86.0 88.3 89.4 90.5 90.9 91.2 91.3 90.2 91.7 90.7 90.9 91.7 92.0 100.0 89.9 91.3
protvec 71.3 86.6 88.6 89.0 89.2 89.8 89.3 89.9 90.0 91.1 90.4 90.9 89.8 91.0 89.9 100.0 90.6
seqvec 70.2 85.7 88.5 89.4 90.2 90.3 89.5 89.9 90.0 91.1 90.8 90.6 90.4 90.1 91.3 90.6 100.0
pfam taap random200 spmap ctriad unirep5700 geary unirep1900 k-sep_pssm apaac dde ctdd transformer-pool qso transformer-avg protvec seqvec
92 91 90 89 88 87 86 85

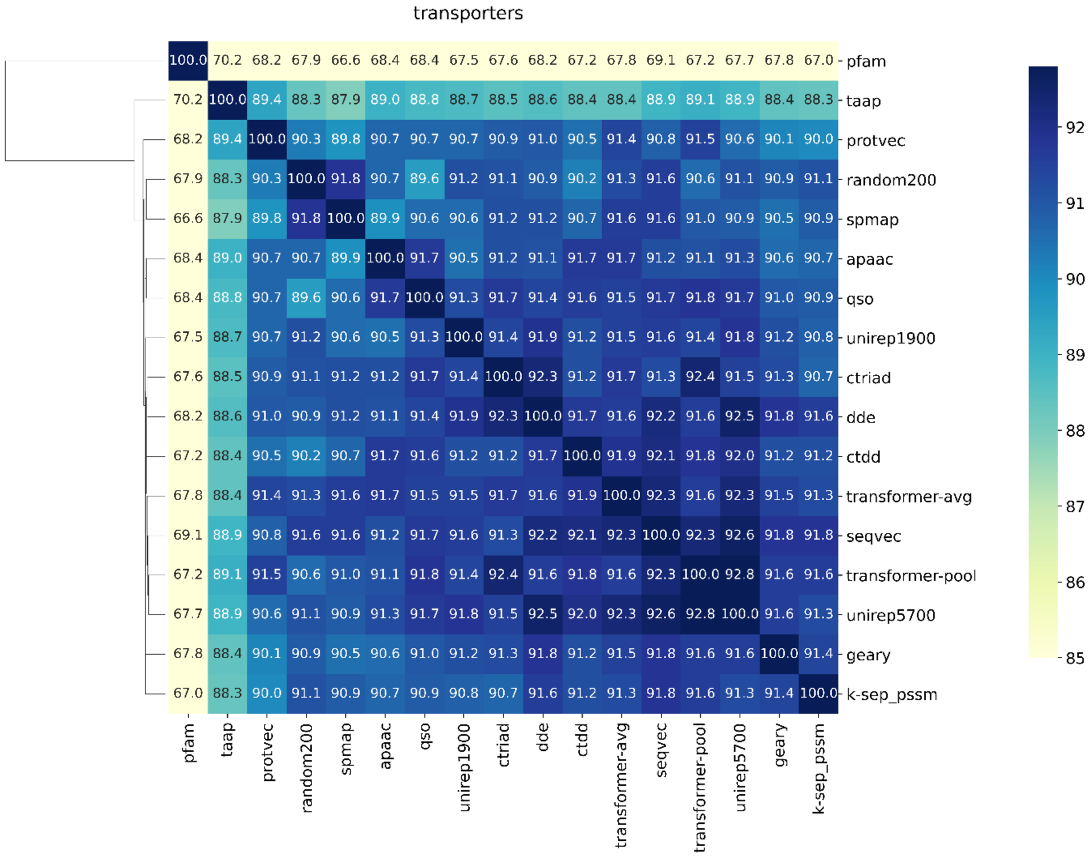
transporters
pfam 100.0 70.2 68.2 67.9 66.6 68.4 68.4 67.5 67.6 68.2 67.2 67.8 69.1 67.2 67.7 67.8 67.0
taap 70.2 100.0 89.4 88.3 87.9 89.0 88.8 88.7 88.5 88.6 88.4 88.4 88.9 89.1 88.9 88.4 88.3
protvec 68.2 89.4 100.0 90.3 89.8 90.7 90.7 90.7 90.9 91.0 90.5 91.4 90.8 91.5 90.6 90.1 90.0
random200 67.9 88.3 90.3 100.0 91.8 90.7 89.6 91.2 91.1 90.9 90.2 91.3 91.6 90.6 91.1 90.9 91.1
spmap 66.6 87.9 89.8 91.8 100.0 89.9 90.6 90.6 91.2 91.2 90.7 91.6 91.6 91.0 90.9 90.5 90.9
apaac 68.4 89.0 90.7 90.7 89.9 100.0 91.7 90.5 91.2 91.1 91.7 91.7 91.2 91.1 91.3 90.6 90.7
qso 68.4 88.8 90.7 89.6 90.6 91.7 100.0 91.3 91.7 91.4 91.6 91.5 91.7 91.8 91.7 91.0 90.9
unirep1900 67.5 88.7 90.7 91.2 90.6 90.5 91.3 100.0 91.4 91.9 91.2 91.5 91.6 91.4 91.8 91.2 90.8
ctriad 67.6 88.5 90.9 91.1 91.2 91.2 91.7 91.4 100.0 92.3 91.2 91.7 91.3 92.4 91.5 91.3 90.7
dde 68.2 88.6 91.0 90.9 91.2 91.1 91.4 91.9 92.3 100.0 91.7 91.6 92.2 91.6 92.5 91.8 91.6
ctdd 67.2 88.4 90.5 90.2 90.7 91.7 91.6 91.2 91.2 91.7 100.0 91.9 92.1 91.8 92.0 91.2 91.2
transformer-avg 67.8 88.4 91.4 91.3 91.6 91.7 91.5 91.5 91.7 91.6 91.9 100.0 92.3 91.6 92.3 91.5 91.3
seqvec 69.1 88.9 90.8 91.6 91.6 91.2 91.7 91.6 91.3 92.2 92.1 92.3 100.0 92.3 92.6 91.8 91.8
transformer-pool 67.2 89.1 91.5 90.6 91.0 91.1 91.8 91.4 92.4 91.6 91.8 91.6 92.3 100.0 92.8 91.6 91.6
unirep5700 67.7 88.9 90.6 91.1 90.9 91.3 91.7 91.8 91.5 92.5 92.0 92.3 92.6 92.8 100.0 91.6 91.3
geary 67.8 88.4 90.1 90.9 90.5 90.6 91.0 91.2 91.3 91.8 91.2 91.5 91.8 91.6 91.6 100.0 91.4
k-sep_pssm 67.0 88.3 90.0 91.1 90.9 90.7 90.9 90.8 90.7 91.6 91.2 91.3 91.8 91.6 91.3 91.4 100.0
pfam taap protvec random200 spmap apaac qso unirep1900 ctriad dde ctdd transformer-avg seqvec transformer-pool unirep5700 geary k-sep_pssm
92 91 90 89 88 87 86 85

(b)

Dissimilar-compound-split

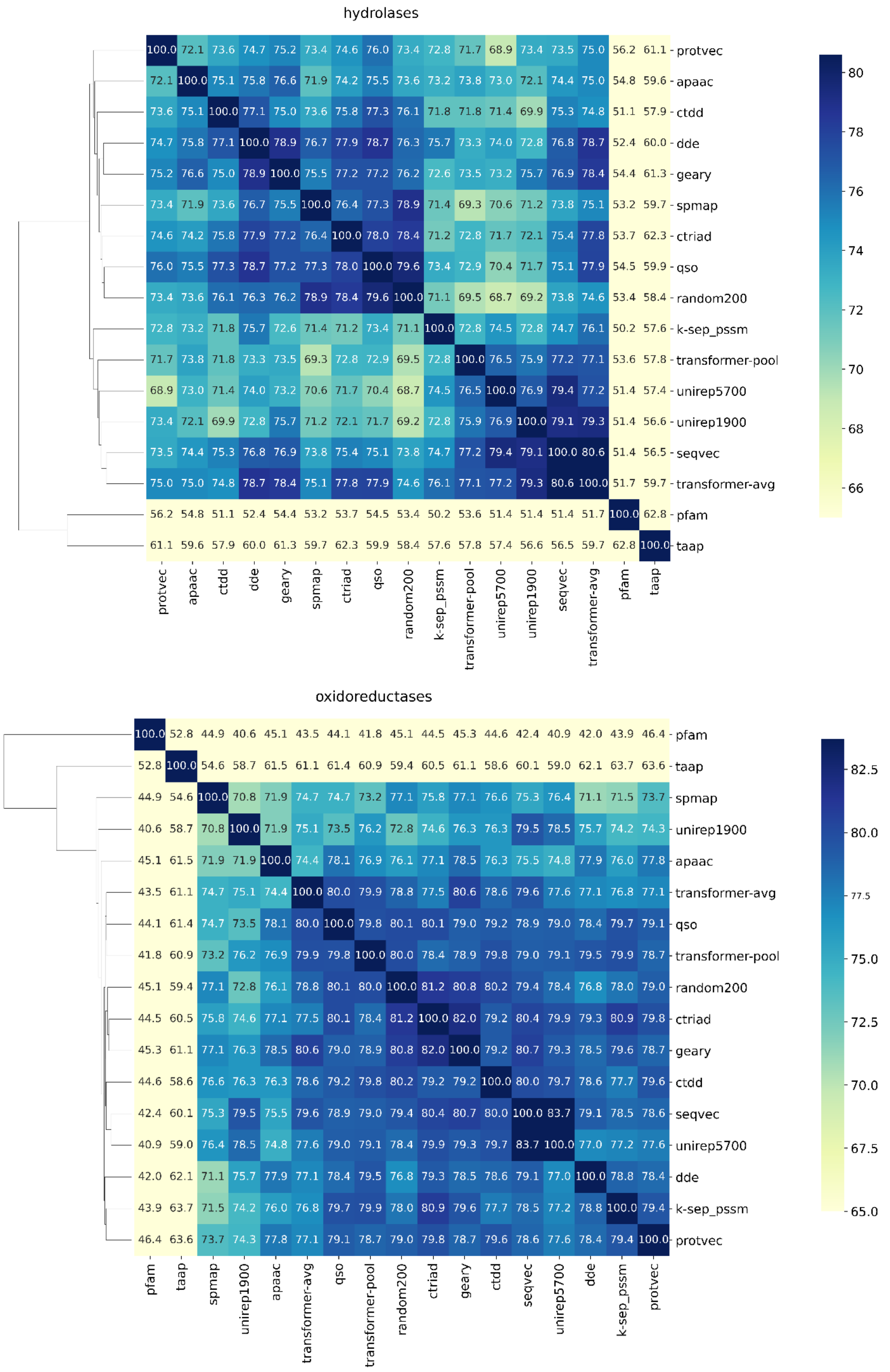

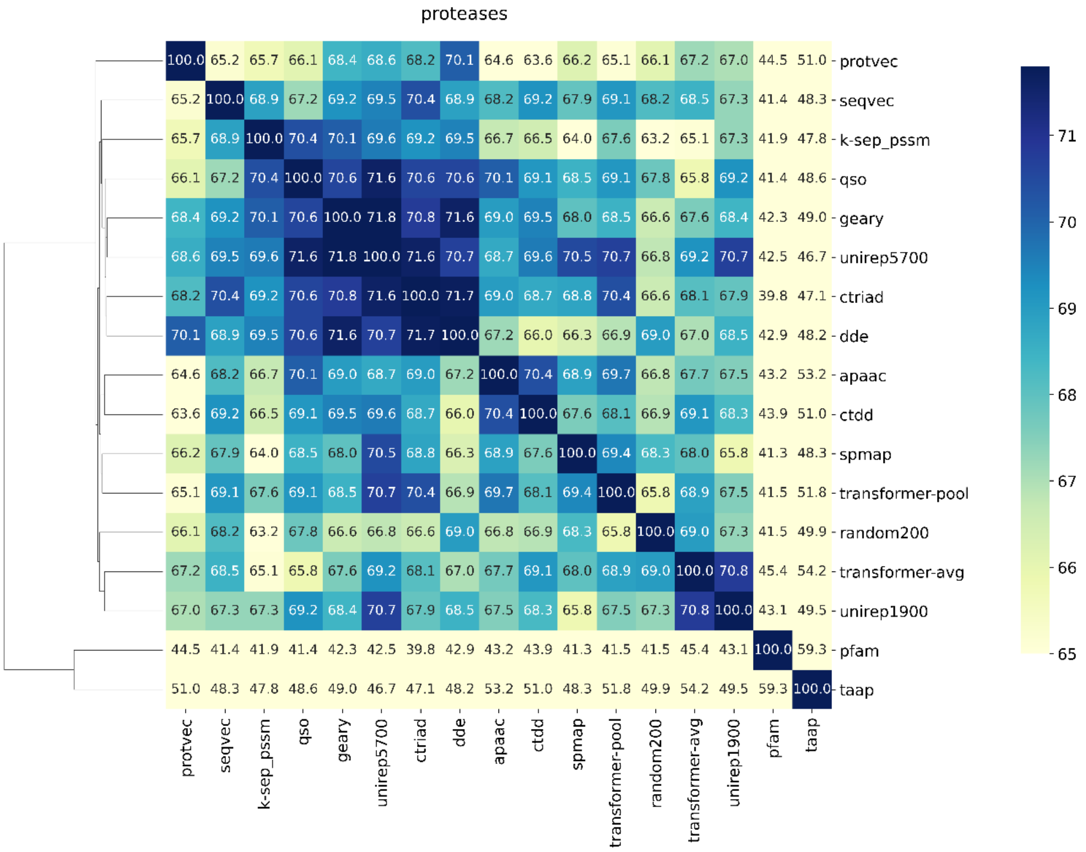
proteases
100.0 65.2 65.7 66.1 68.4 68.6 68.2 70.1 64.6 63.6 66.2 65.1 66.1 67.2 67.0 44.5 51.0 protvec
65.2 100.0 68.9 67.2 69.2 69.5 70.4 68.9 68.2 69.2 67.9 69.1 68.2 68.5 67.3 41.4 48.3 seqvec
65.7 68.9 100.0 70.4 70.1 69.6 69.2 69.5 66.7 66.5 64.0 67.6 63.2 65.1 67.3 41.9 47.8 k-sep_pssm
66.1 67.2 70.4 100.0 70.6 71.6 70.6 70.6 70.1 69.1 68.5 69.1 67.8 65.8 69.2 41.4 48.6 qso
68.4 69.2 70.1 70.6 100.0 71.8 70.8 71.6 69.0 69.5 68.0 68.5 66.6 67.6 68.4 42.3 49.0 geary
68.6 69.5 69.6 71.6 71.8 100.0 71.6 70.7 68.7 69.6 70.5 70.7 66.8 69.2 70.7 42.5 46.7 unirep5700
68.2 70.4 69.2 70.6 70.8 71.6 100.0 71.7 69.0 68.7 68.8 70.4 66.6 68.1 67.9 39.8 47.1 ctriad
70.1 68.9 69.5 70.6 71.6 70.7 71.7 100.0 67.2 66.0 66.3 66.9 69.0 67.0 68.5 42.9 48.2 dde
64.6 68.2 66.7 70.1 69.0 68.7 69.0 67.2 100.0 70.4 68.9 69.7 66.8 67.7 67.5 43.2 53.2 apaac
63.6 69.2 66.5 69.1 69.5 69.6 68.7 66.0 70.4 100.0 67.6 68.1 66.9 69.1 68.3 43.9 51.0 ctdd
66.2 67.9 64.0 68.5 68.0 70.5 68.8 66.3 68.9 67.6 100.0 69.4 68.3 68.0 65.8 41.3 48.3 spmap
65.1 69.1 67.6 69.1 68.5 70.7 70.4 66.9 69.7 68.1 69.4 100.0 65.8 68.9 67.5 41.5 51.8 transformer-pool
66.1 68.2 63.2 67.8 66.6 66.8 66.6 69.0 66.8 66.9 68.3 65.8 100.0 69.0 67.3 41.5 49.9 random200
67.2 68.5 65.1 65.8 67.6 69.2 68.1 67.0 67.7 69.1 68.0 68.9 69.0 100.0 70.8 45.4 54.2 transformer-avg
67.0 67.3 67.3 69.2 68.4 70.7 67.9 68.5 67.5 68.3 65.8 67.5 67.3 70.8 100.0 43.1 49.5 unirep1900
44.5 41.4 41.9 41.4 42.3 42.5 39.8 42.9 43.2 43.9 41.3 41.5 41.5 45.4 43.1 100.0 59.3 pfam
51.0 48.3 47.8 48.6 49.0 46.7 47.1 48.2 53.2 51.0 48.3 51.8 49.9 54.2 49.5 59.3 100.0 taap
protvec
seqvec
k-sep_pssm
qso
geary
unirep5700
ctriad
dde
apaac
ctdd
spmap
transformer-pool
random200
transformer-avg
unirep1900
pfam
taap
71
70
69
68
67
66
65

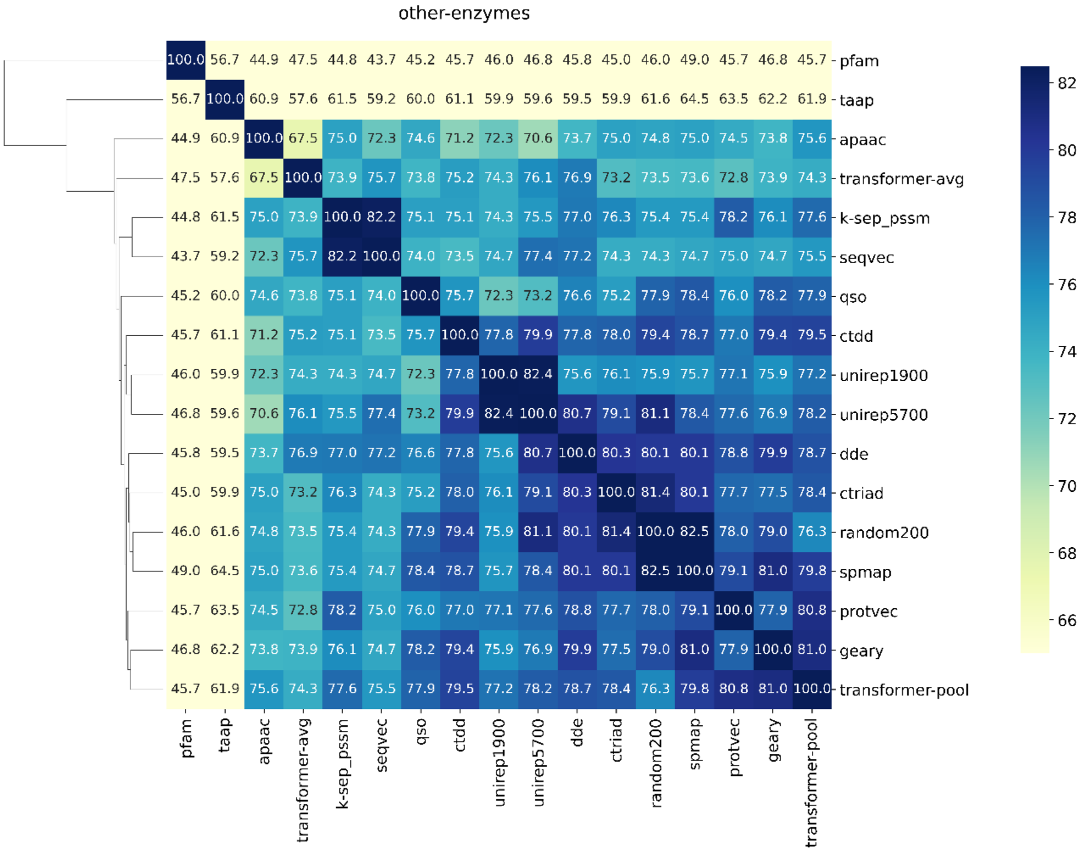
other-enzymes
100.0 56.7 44.9 47.5 44.8 43.7 45.2 45.7 46.0 46.8 45.8 45.0 46.0 49.0 45.7 46.8 45.7 pfam
56.7 100.0 60.9 57.6 61.5 59.2 60.0 61.1 59.9 59.6 59.5 59.9 61.6 64.5 63.5 62.2 61.9 taap
44.9 60.9 100.0 67.5 75.0 72.3 74.6 71.2 72.3 70.6 73.7 75.0 74.8 75.0 74.5 73.8 75.6 apaac
47.5 57.6 67.5 100.0 73.9 75.7 73.8 75.2 74.3 76.1 76.9 73.2 73.5 73.6 72.8 73.9 74.3 transformer-avg
44.8 61.5 75.0 73.9 100.0 82.2 75.1 75.1 74.3 75.5 77.0 76.3 75.4 75.4 78.2 76.1 77.6 k-sep_pssm
43.7 59.2 72.3 75.7 82.2 100.0 74.0 73.5 74.7 77.4 77.2 74.3 74.3 74.7 75.0 74.7 75.5 seqvec
45.2 60.0 74.6 73.8 75.1 74.0 100.0 75.7 72.3 73.2 76.6 75.2 77.9 78.4 76.0 78.2 77.9 qso
45.7 61.1 71.2 75.2 75.1 73.5 75.7 100.0 77.8 79.9 77.8 78.0 79.4 78.7 77.0 79.4 79.5 ctdd
46.0 59.9 72.3 74.3 74.3 74.7 72.3 77.8 100.0 82.4 75.6 76.1 75.9 75.7 77.1 75.9 77.2 unirep1900
46.8 59.6 70.6 76.1 75.5 77.4 73.2 79.9 82.4 100.0 80.7 79.1 81.1 78.4 77.6 76.9 78.2 unirep5700
45.8 59.5 73.7 76.9 77.0 77.2 76.6 77.8 75.6 80.7 100.0 80.3 80.1 80.1 78.8 79.9 78.7 dde
45.0 59.9 75.0 73.2 76.3 74.3 75.2 78.0 76.1 79.1 80.3 100.0 81.4 80.1 77.7 77.5 78.4 ctriad
46.0 61.6 74.8 73.5 75.4 74.3 77.9 79.4 75.9 81.1 80.1 81.4 100.0 82.5 78.0 79.0 76.3 random200
49.0 64.5 75.0 73.6 75.4 74.7 78.4 78.7 75.7 78.4 80.1 80.1 82.5 100.0 79.1 81.0 79.8 spmap
45.7 63.5 74.5 72.8 78.2 75.0 76.0 77.0 77.1 77.6 78.8 77.7 78.0 79.1 100.0 77.9 80.8 protvec
46.8 62.2 73.8 73.9 76.1 74.7 78.2 79.4 75.9 76.9 79.9 77.5 79.0 81.0 77.9 100.0 81.0 geary
45.7 61.9 75.6 74.3 77.6 75.5 77.9 79.5 77.2 78.2 78.7 78.4 76.3 79.8 80.8 81.0 100.0 transformer-pool
pfam
taap
apaac
transformer-avg
k-sep_pssm
seqvec
qso
ctdd
unirep1900
unirep5700
dde
ctriad
random200
spmap
protvec
geary
transformer-pool
82
80
78
76
74
72
70
68
66

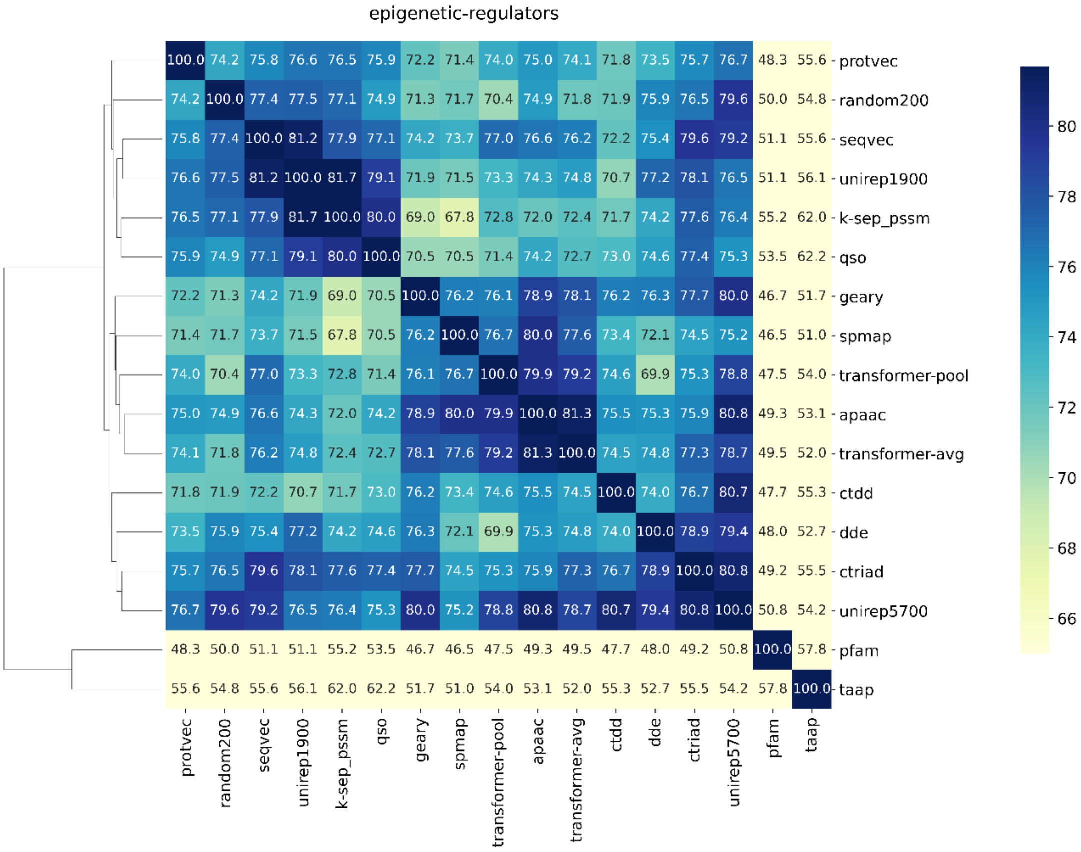
epigenetic-regulators
protvec 100.0 74.2 75.8 76.6 76.5 75.9 72.2 71.4 74.0 75.0 74.1 71.8 73.5 75.7 76.7 48.3 55.6
random200 74.2 100.0 77.4 77.5 77.1 74.9 71.3 71.7 70.4 74.9 71.8 71.9 75.9 76.5 79.6 50.0 54.8
seqvec 75.8 77.4 100.0 81.2 77.9 77.1 74.2 73.7 77.0 76.6 76.2 72.2 75.4 79.6 79.2 51.1 55.6
unirep1900 76.6 77.5 81.2 100.0 81.7 79.1 71.9 71.5 73.3 74.3 74.8 70.7 77.2 78.1 76.5 51.1 56.1
k-sep_pssm 76.5 77.1 77.9 81.7 100.0 80.0 69.0 67.8 72.8 72.0 72.4 71.7 74.2 77.6 76.4 55.2 62.0
qso 75.9 74.9 77.1 79.1 80.0 100.0 70.5 70.5 71.4 74.2 72.7 73.0 74.6 77.4 75.3 53.5 62.2
geary 72.2 71.3 74.2 71.9 69.0 70.5 100.0 76.2 76.1 78.9 78.1 76.2 76.3 77.7 80.0 46.7 51.7
spmap 71.4 71.7 73.7 71.5 67.8 70.5 76.2 100.0 76.7 80.0 77.6 73.4 72.1 74.5 75.2 46.5 51.0
transformer-pool 74.0 70.4 77.0 73.3 72.8 71.4 76.1 76.7 100.0 79.9 79.2 74.6 69.9 75.3 78.8 47.5 54.0
apaac 75.0 74.9 76.6 74.3 72.0 74.2 78.9 80.0 79.9 100.0 81.3 75.5 75.3 75.9 80.8 49.3 53.1
transformer-avg 74.1 71.8 76.2 74.8 72.4 72.7 78.1 77.6 79.2 81.3 100.0 74.5 74.8 77.3 78.7 49.5 52.0
ctdd 71.8 71.9 72.2 70.7 71.7 73.0 76.2 73.4 74.6 75.5 74.5 100.0 74.0 76.7 80.7 47.7 55.3
dde 73.5 75.9 75.4 77.2 74.2 74.6 76.3 72.1 69.9 75.3 74.8 74.0 100.0 78.9 79.4 48.0 52.7
ctriad 75.7 76.5 79.6 78.1 77.6 77.4 77.7 74.5 75.3 75.9 77.3 76.7 78.9 100.0 80.8 49.2 55.5
unirep5700 76.7 79.6 79.2 76.5 76.4 75.3 80.0 75.2 78.8 80.8 78.7 80.7 79.4 80.8 100.0 50.8 54.2
pfam 48.3 50.0 51.1 51.1 55.2 53.5 46.7 46.5 47.5 49.3 49.5 47.7 48.0 49.2 50.8 100.0 57.8
taap 55.6 54.8 55.6 56.1 62.0 62.2 51.7 51.0 54.0 53.1 52.0 55.3 52.7 55.5 54.2 57.8 100.0
protvec random200 seqvec unirep1900 k-sep_pssm qso geary spmap transformer-pool apaac transformer-avg ctdd dde ctriad unirep5700 pfam taap
80 78 76 74 72 70 68 66

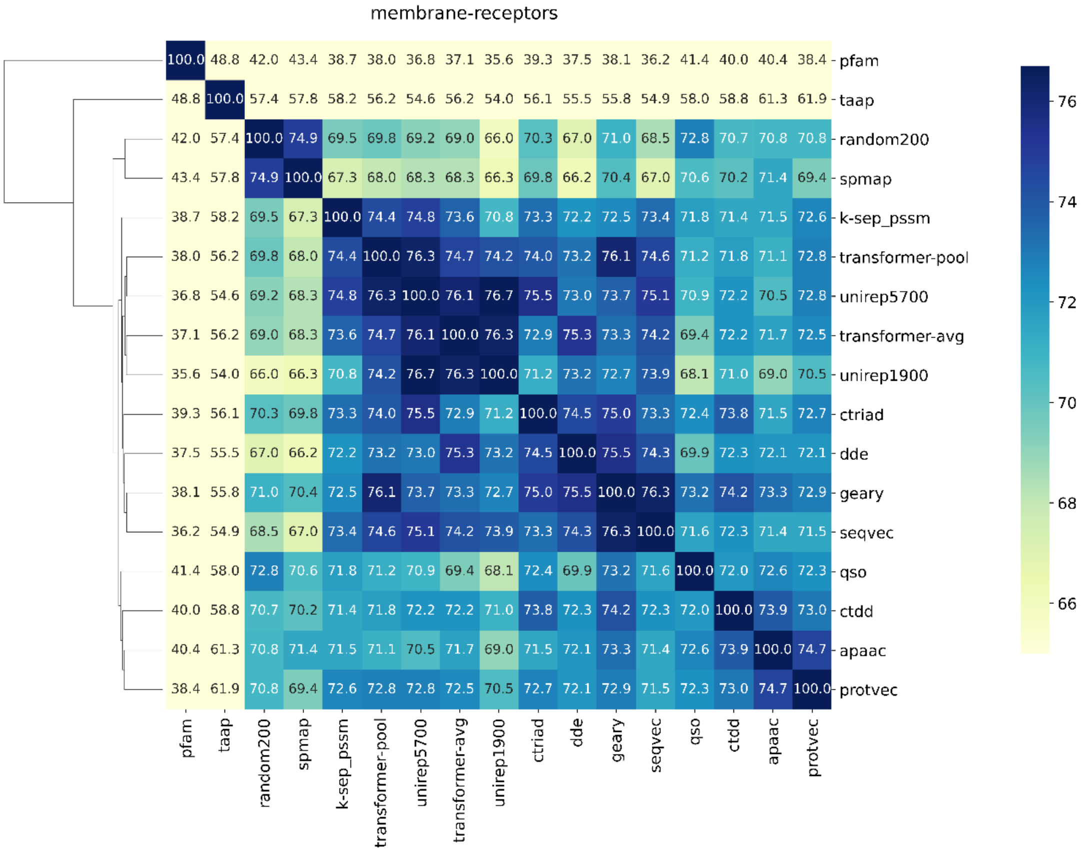
membrane-receptors
pfam 100.0 48.8 42.0 43.4 38.7 38.0 36.8 37.1 35.6 39.3 37.5 38.1 36.2 41.4 40.0 40.4 38.4
taap 48.8 100.0 57.4 57.8 58.2 56.2 54.6 56.2 54.0 56.1 55.5 55.8 54.9 58.0 58.8 61.3 61.9
random200 42.0 57.4 100.0 74.9 69.5 69.8 69.2 69.0 66.0 70.3 67.0 71.0 68.5 72.8 70.7 70.8 70.8
spmap 43.4 57.8 74.9 100.0 67.3 68.0 68.3 68.3 66.3 69.8 66.2 70.4 67.0 70.6 70.2 71.4 69.4
k-sep_pssm 38.7 58.2 69.5 67.3 100.0 74.4 74.8 73.6 70.8 73.3 72.2 72.5 73.4 71.8 71.4 71.5 72.6
transformer-pool 38.0 56.2 69.8 68.0 74.4 100.0 76.3 74.7 74.2 74.0 73.2 76.1 74.6 71.2 71.8 71.1 72.8
unirep5700 36.8 54.6 69.2 68.3 74.8 76.3 100.0 76.1 76.7 75.5 73.0 73.7 75.1 70.9 72.2 70.5 72.8
transformer-avg 37.1 56.2 69.0 68.3 73.6 74.7 76.1 100.0 76.3 72.9 75.3 73.3 74.2 69.4 72.2 71.7 72.5
unirep1900 35.6 54.0 66.0 66.3 70.8 74.2 76.7 76.3 100.0 71.2 73.2 72.7 73.9 68.1 71.0 69.0 70.5
ctriad 39.3 56.1 70.3 69.8 73.3 74.0 75.5 72.9 71.2 100.0 74.5 75.0 73.3 72.4 73.8 71.5 72.7
dde 37.5 55.5 67.0 66.2 72.2 73.2 73.0 75.3 73.2 74.5 100.0 75.5 74.3 69.9 72.3 72.1 72.1
geary 38.1 55.8 71.0 70.4 72.5 76.1 73.7 73.3 72.7 75.0 75.5 100.0 76.3 73.2 74.2 73.3 72.9
seqvec 36.2 54.9 68.5 67.0 73.4 74.6 75.1 74.2 73.9 73.3 74.3 76.3 100.0 71.6 72.3 71.4 71.5
qso 41.4 58.0 72.8 70.6 71.8 71.2 70.9 69.4 68.1 72.4 69.9 73.2 71.6 100.0 72.0 72.6 72.3
ctdd 40.0 58.8 70.7 70.2 71.4 71.8 72.2 72.2 71.0 73.8 72.3 74.2 72.3 72.0 100.0 73.9 73.0
apaac 40.4 61.3 70.8 71.4 71.5 71.1 70.5 71.7 69.0 71.5 72.1 73.3 71.4 72.6 73.9 100.0 74.7
protvec 38.4 61.9 70.8 69.4 72.6 72.8 72.8 72.5 70.5 72.7 72.1 72.9 71.5 72.3 73.0 74.7 100.0
pfam taap random200 spmap k-sep_pssm transformer-pool unirep5700 transformer-avg unirep1900 ctriad dde geary seqvec qso ctdd apaac protvec
76 74 72 70 68 66

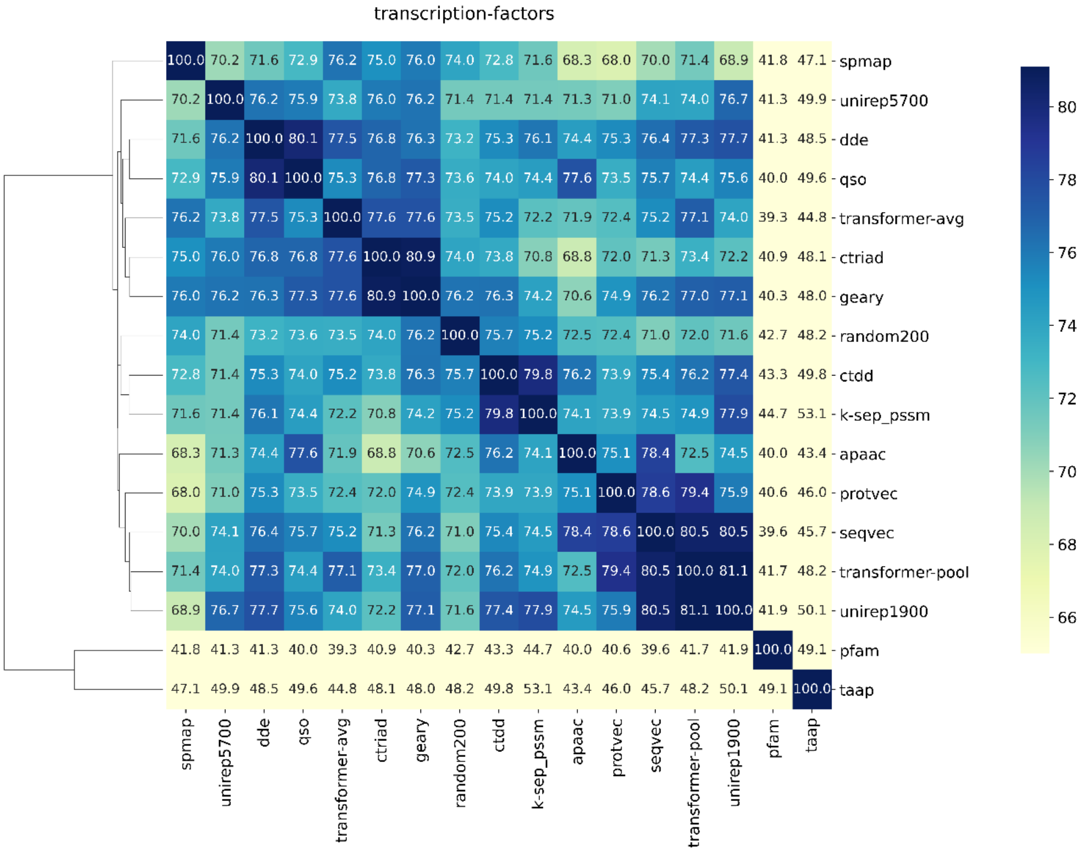
transcription-factors
spmap
unirep5700
dde
qso
transformer-avg
ctriad
geary
random200
ctdd
k-sep_pssm
apaac
protvec
seqvec
transformer-pool
unirep1900
pfam
taap
80
78
76
74
72
70
68
66

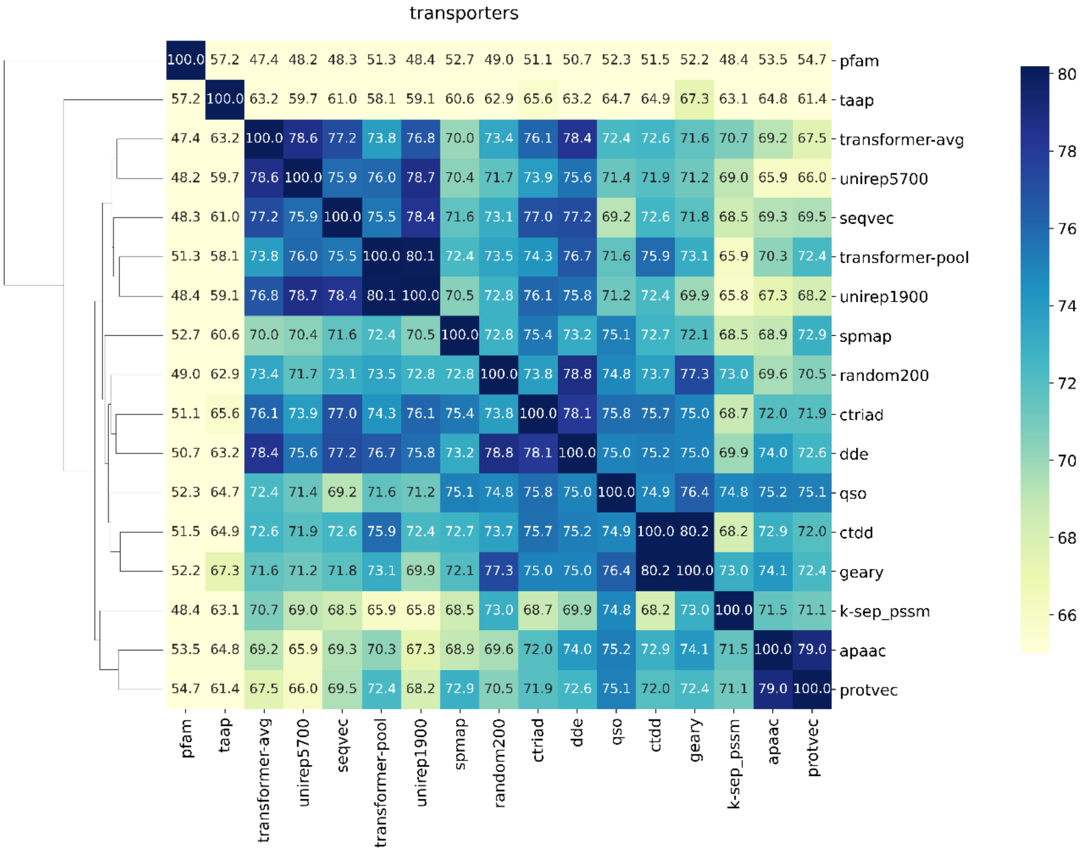
transporters
pfam
taap
transformer-avg
unirep5700
seqvec
transformer-pool
unirep1900
spmap
random200
ctriad
dde
qso
ctdd
geary
k-sep_pssm
apaac
protvec
80
78
76
74
72
70
68
66

(c)

Fully-dissimilar-split

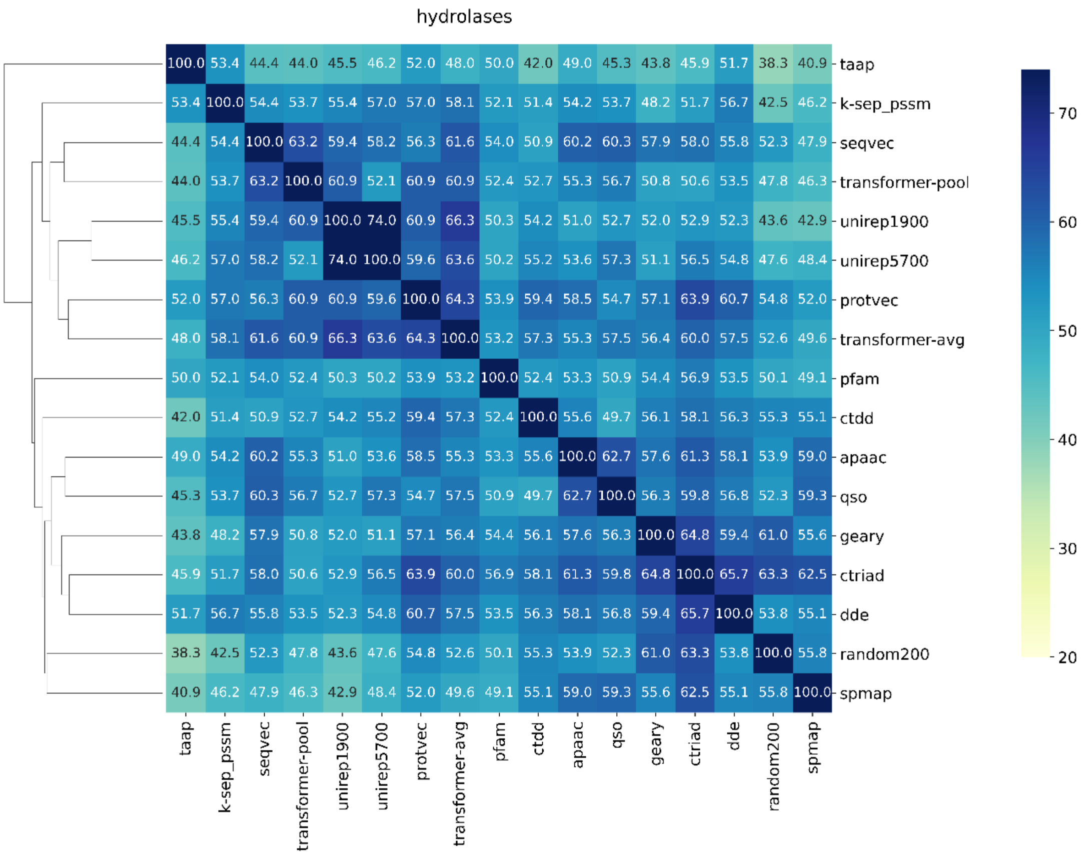

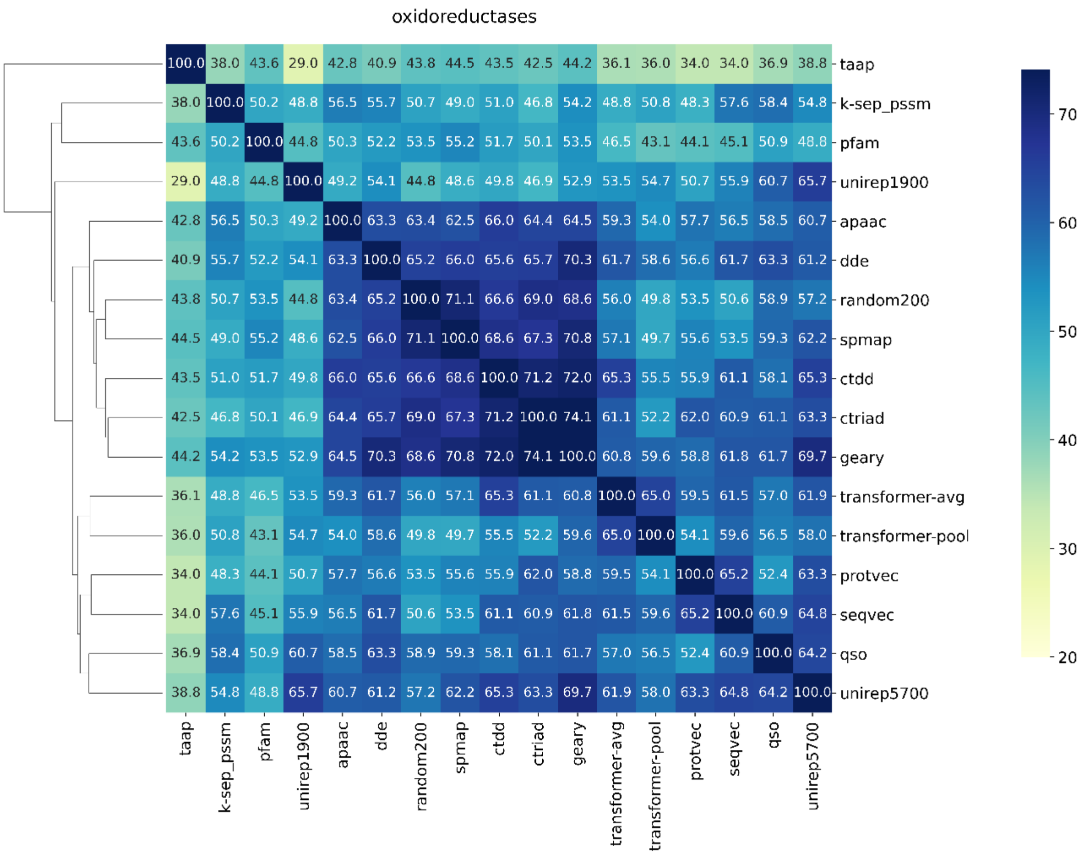

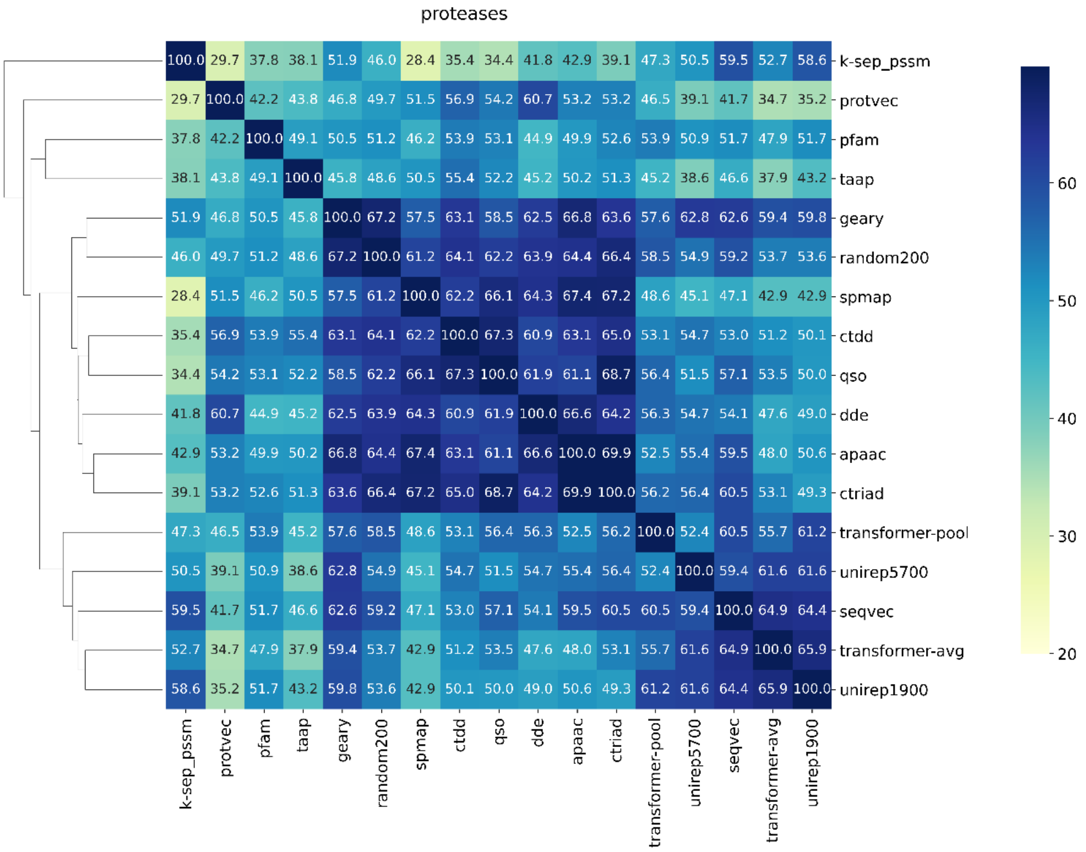
proteases
k-sep_pssm
protvec
pfam
taap
geary
random200
spmap
ctdd
qso
dde
apaac
ctriad
transformer-pool
unirep5700
seqvec
transformer-avg
unirep1900
60
50
40
30
20

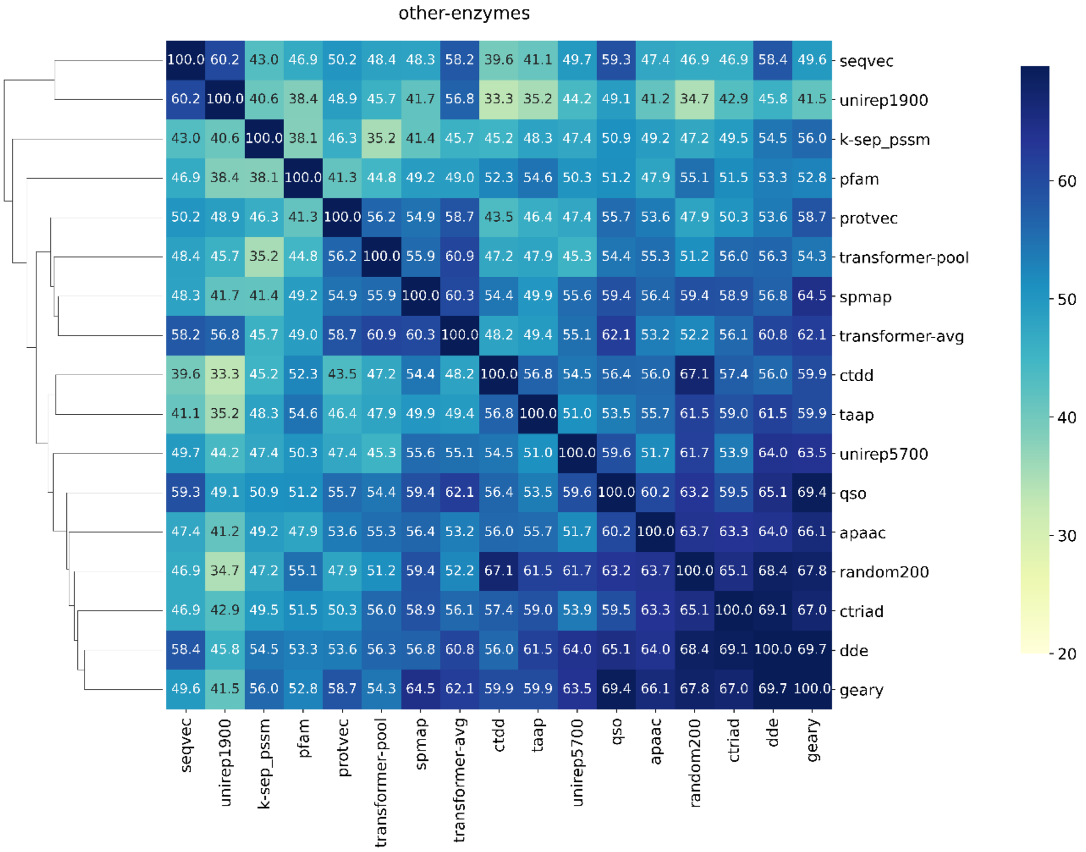
other-enzymes
seqvec
unirep1900
k-sep_pssm
pfam
protvec
transformer-pool
spmap
transformer-avg
ctdd
taap
unirep5700
qso
apaac
random200
ctriad
dde
geary
60
50
40
30
20

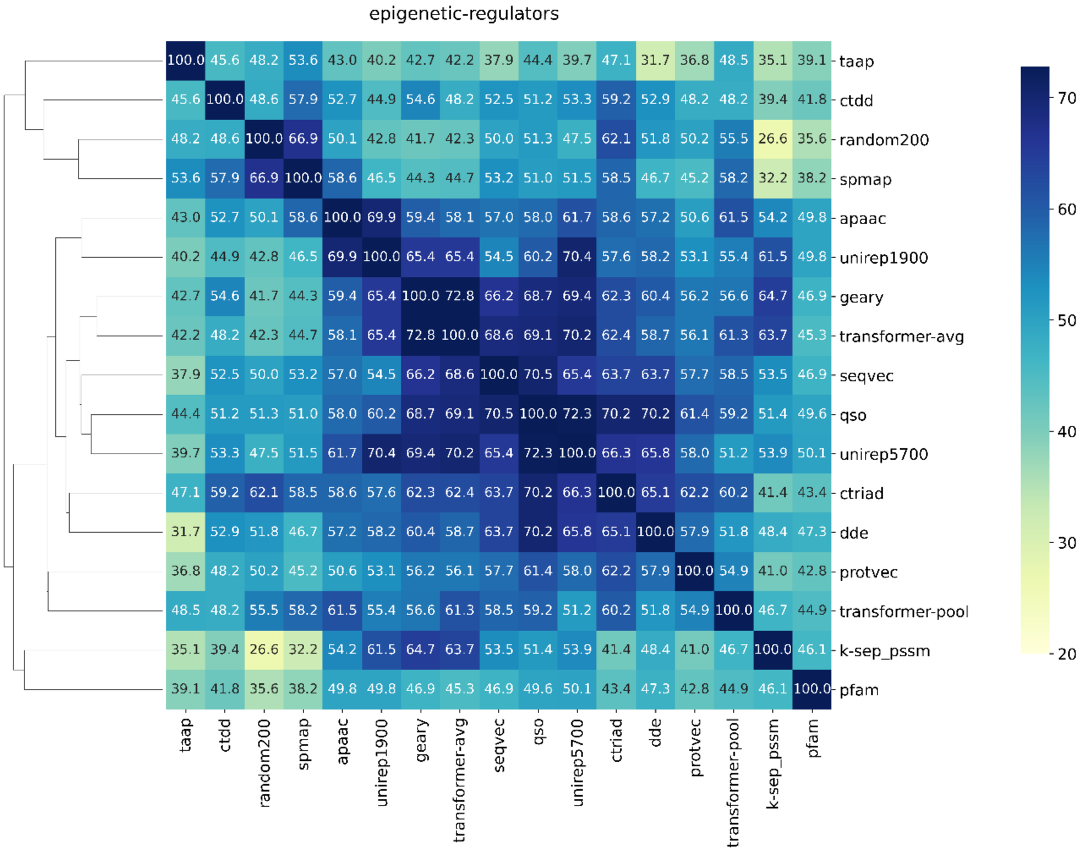
epigenetic-regulators
taap 100.0 45.6 48.2 53.6 43.0 40.2 42.7 42.2 37.9 44.4 39.7 47.1 31.7 36.8 48.5 35.1 39.1
ctdd 45.6 100.0 48.6 57.9 52.7 44.9 54.6 48.2 52.5 51.2 53.3 59.2 52.9 48.2 48.2 39.4 41.8
random200 48.2 48.6 100.0 66.9 50.1 42.8 41.7 42.3 50.0 51.3 47.5 62.1 51.8 50.2 55.5 26.6 35.6
spmap 53.6 57.9 66.9 100.0 58.6 46.5 44.3 44.7 53.2 51.0 51.5 58.5 46.7 45.2 58.2 32.2 38.2
apaac 43.0 52.7 50.1 58.6 100.0 69.9 59.4 58.1 57.0 58.0 61.7 58.6 57.2 50.6 61.5 54.2 49.8
unirep1900 40.2 44.9 42.8 46.5 69.9 100.0 65.4 65.4 54.5 60.2 70.4 57.6 58.2 53.1 55.4 61.5 49.8
geary 42.7 54.6 41.7 44.3 59.4 65.4 100.0 72.8 66.2 68.7 69.4 62.3 60.4 56.2 56.6 64.7 46.9
transformer-avg 42.2 48.2 42.3 44.7 58.1 65.4 72.8 100.0 68.6 69.1 70.2 62.4 58.7 56.1 61.3 63.7 45.3
seqvec 37.9 52.5 50.0 53.2 57.0 54.5 66.2 68.6 100.0 70.5 65.4 63.7 63.7 57.7 58.5 53.5 46.9
qso 44.4 51.2 51.3 51.0 58.0 60.2 68.7 69.1 70.5 100.0 72.3 70.2 70.2 61.4 59.2 51.4 49.6
unirep5700 39.7 53.3 47.5 51.5 61.7 70.4 69.4 70.2 65.4 72.3 100.0 66.3 65.8 58.0 51.2 53.9 50.1
ctriad 47.1 59.2 62.1 58.5 58.6 57.6 62.3 62.4 63.7 70.2 66.3 100.0 65.1 62.2 60.2 41.4 43.4
dde 31.7 52.9 51.8 46.7 57.2 58.2 60.4 58.7 63.7 70.2 65.8 65.1 100.0 57.9 51.8 48.4 47.3
protvec 36.8 48.2 50.2 45.2 50.6 53.1 56.2 56.1 57.7 61.4 58.0 62.2 57.9 100.0 54.9 41.0 42.8
transformer-pool 48.5 48.2 55.5 58.2 61.5 55.4 56.6 61.3 58.5 59.2 51.2 60.2 51.8 54.9 100.0 46.7 44.9
k-sep_pssm 35.1 39.4 26.6 32.2 54.2 61.5 64.7 63.7 53.5 51.4 53.9 41.4 48.4 41.0 46.7 100.0 46.1
pfam 39.1 41.8 35.6 38.2 49.8 49.8 46.9 45.3 46.9 49.6 50.1 43.4 47.3 42.8 44.9 46.1 100.0
taap ctdd random200 spmap apaac unirep1900 geary transformer-avg seqvec qso unirep5700 ctriad dde protvec transformer-pool k-sep_pssm pfam
70
60
50
40
30
20

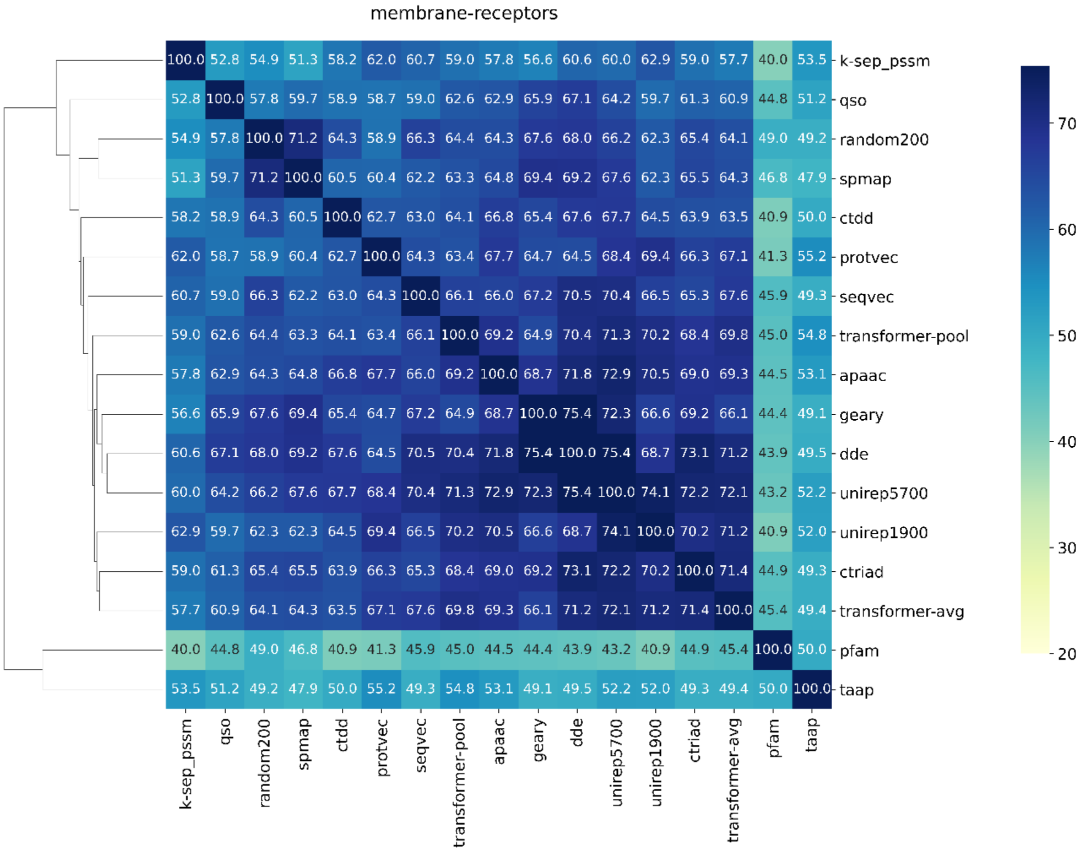
membrane-receptors
k-sep_pssm 100.0 52.8 54.9 51.3 58.2 62.0 60.7 59.0 57.8 56.6 60.6 60.0 62.9 59.0 57.7 40.0 53.5
qso 52.8 100.0 57.8 59.7 58.9 58.7 59.0 62.6 62.9 65.9 67.1 64.2 59.7 61.3 60.9 44.8 51.2
random200 54.9 57.8 100.0 71.2 64.3 58.9 66.3 64.4 64.3 67.6 68.0 66.2 62.3 65.4 64.1 49.0 49.2
spmap 51.3 59.7 71.2 100.0 60.5 60.4 62.2 63.3 64.8 69.4 69.2 67.6 62.3 65.5 64.3 46.8 47.9
ctdd 58.2 58.9 64.3 60.5 100.0 62.7 63.0 64.1 66.8 65.4 67.6 67.7 64.5 63.9 63.5 40.9 50.0
protvec 62.0 58.7 58.9 60.4 62.7 100.0 64.3 63.4 67.7 64.7 64.5 68.4 69.4 66.3 67.1 41.3 55.2
seqvec 60.7 59.0 66.3 62.2 63.0 64.3 100.0 66.1 66.0 67.2 70.5 70.4 66.5 65.3 67.6 45.9 49.3
transformer-pool 59.0 62.6 64.4 63.3 64.1 63.4 66.1 100.0 69.2 64.9 70.4 71.3 70.2 68.4 69.8 45.0 54.8
apaac 57.8 62.9 64.3 64.8 66.8 67.7 66.0 69.2 100.0 68.7 71.8 72.9 70.5 69.0 69.3 44.5 53.1
geary 56.6 65.9 67.6 69.4 65.4 64.7 67.2 64.9 68.7 100.0 75.4 72.3 66.6 69.2 66.1 44.4 49.1
dde 60.6 67.1 68.0 69.2 67.6 64.5 70.5 70.4 71.8 75.4 100.0 75.4 68.7 73.1 71.2 43.9 49.5
unirep5700 60.0 64.2 66.2 67.6 67.7 68.4 70.4 71.3 72.9 72.3 75.4 100.0 74.1 72.2 72.1 43.2 52.2
unirep1900 62.9 59.7 62.3 62.3 64.5 69.4 66.5 70.2 70.5 66.6 68.7 74.1 100.0 70.2 71.2 40.9 52.0
ctriad 59.0 61.3 65.4 65.5 63.9 66.3 65.3 68.4 69.0 69.2 73.1 72.2 70.2 100.0 71.4 44.9 49.3
transformer-avg 57.7 60.9 64.1 64.3 63.5 67.1 67.6 69.8 69.3 66.1 71.2 72.1 71.2 71.4 100.0 45.4 49.4
pfam 40.0 44.8 49.0 46.8 40.9 41.3 45.9 45.0 44.5 44.4 43.9 43.2 40.9 44.9 45.4 100.0 50.0
taap 53.5 51.2 49.2 47.9 50.0 55.2 49.3 54.8 53.1 49.1 49.5 52.2 52.0 49.3 49.4 50.0 100.0
k-sep_pssm qso random200 spmap ctdd protvec seqvec transformer-pool apaac geary dde unirep5700 unirep1900 ctriad transformer-avg pfam taap
70
60
50
40
30
20

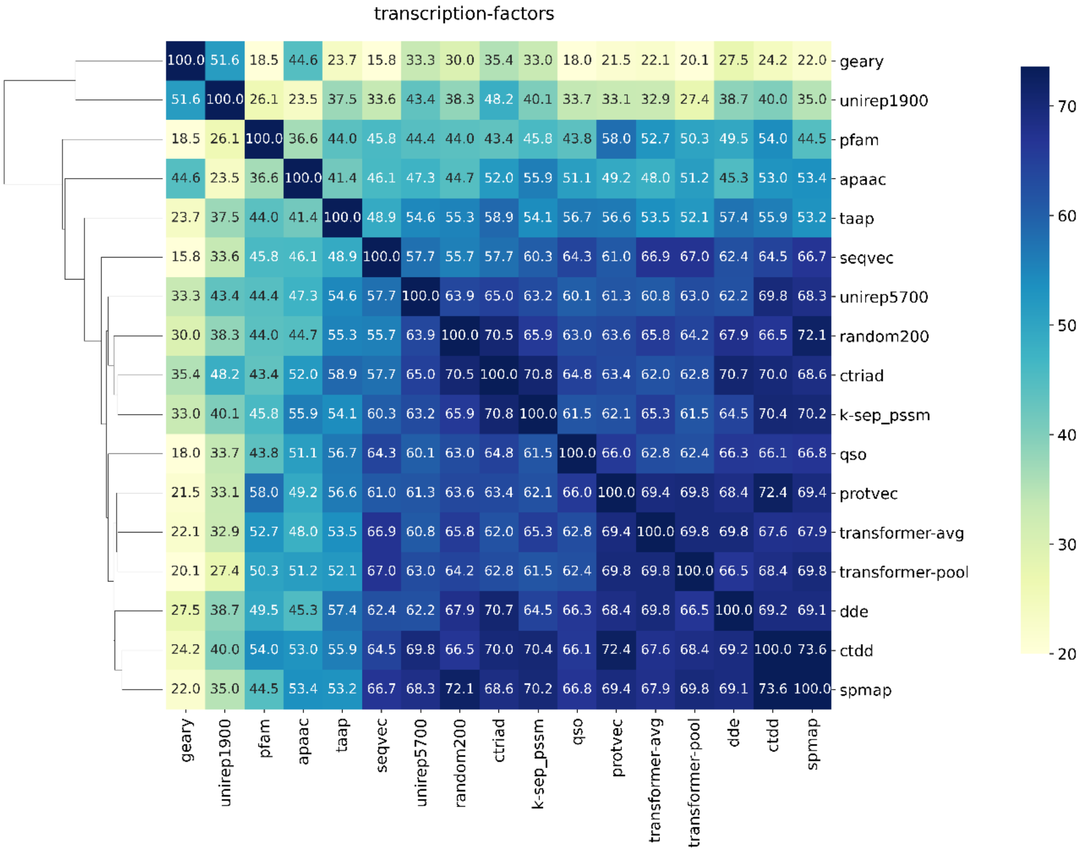

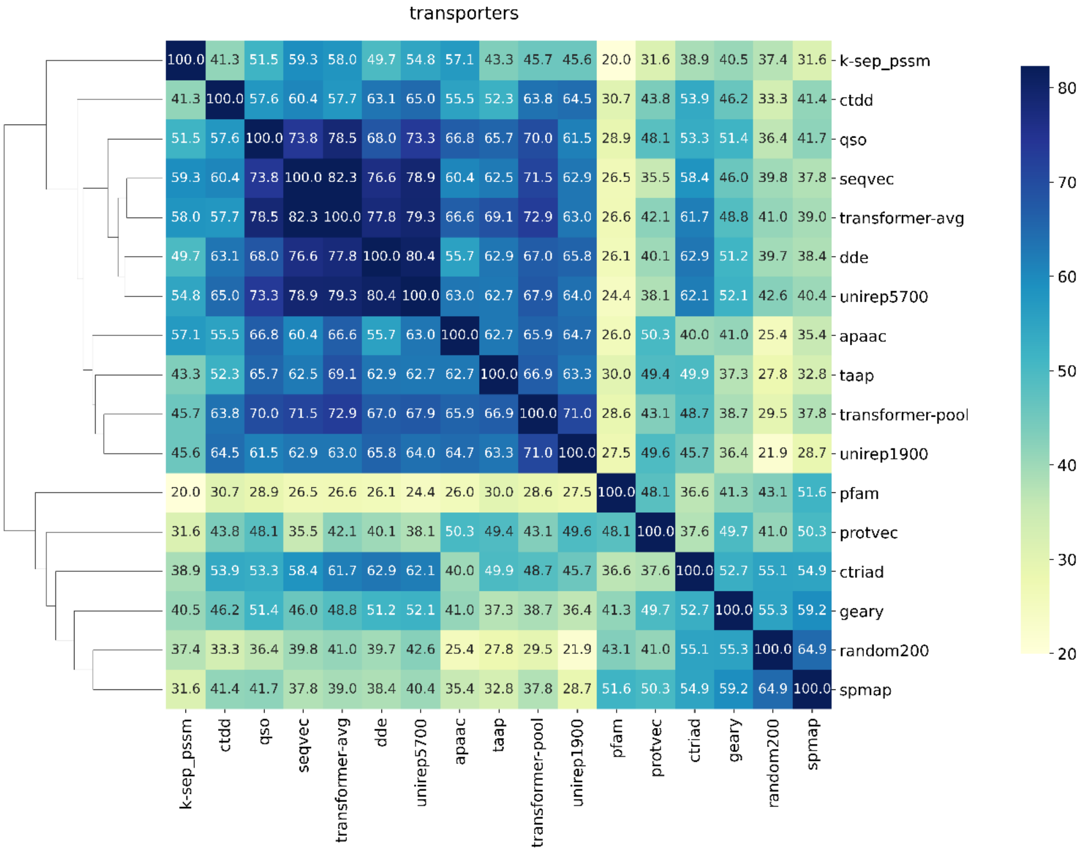

Figure S4. Clustered heatmaps of different protein representation approaches for protein families on **(a)** the random-split, **(b)** dissimilar-compound-split, and **(c)** fully-dissimilar-split datasets.
